# Recent Advances Regarding the Molecular Mechanisms of Triterpenic Acids: A Review (Part II)

**DOI:** 10.3390/ijms23168896

**Published:** 2022-08-10

**Authors:** Marius Mioc, Alexandra Prodea, Roxana Racoviceanu, Alexandra Mioc, Roxana Ghiulai, Andreea Milan, Mirela Voicu, Gabriel Mardale, Codruța Șoica

**Affiliations:** 1 Department of Pharmaceutical Chemistry, Faculty of Pharmacy, Victor Babes University of Medicine and Pharmacy, 2nd Eftimie Murgu Sq., 300041 Timisoara, Romania; 2 Research Centre for Pharmaco-Toxicological Evaluation, “Victor Babes” University of Medicine and Pharmacy, Eftimie Murgu Sq., No. 2, 300041 Timisoara, Romania; 3 Department of Anatomy, Physiology, Pathophysiology, Faculty of Pharmacy, “Victor Babes” University of Medicine and Pharmacy, Eftimie Murgu Sq., No. 2, 300041 Timisoara, Romania; 4 Department of Pharmacology-Pharmacotherapy, Faculty of Pharmacy, Victor Babes University of Medicine and Pharmacy, 2nd Eftimie Murgu Sq., 300041 Timisoara, Romania

**Keywords:** molecular mechanism, triterpenic acids, apoptosis, betulinic acid, glycyrrhetinic acid, maslinic acid, boswellic acid, madecassic acid, pomolic acid, moronic acid, pentacyclic triterpenes

## Abstract

Triterpenic acids are a widespread class of phytocompounds which have been found to possess valuable therapeutic properties such as anticancer, anti-inflammatory, hepatoprotective, cardioprotective, antidiabetic, neuroprotective, lipolytic, antiviral, and antiparasitic effects. They are a subclass of triterpenes bearing a characteristic lipophilic structure that imprints unfavorable in vivo properties which subsequently limit their applications. The early investigation of the mechanism of action (MOA) of a drug candidate can provide valuable information regarding the possible side effects and drug interactions that may occur after administration. The current paper aimed to summarize the most recent (last 5 years) studies regarding the MOA of betulinic acid, boswellic acid, glycyrrhetinic acid, madecassic acid, moronic acid, and pomolic acid in order to provide scientists with updated and accessible material on the topic that could contribute to the development of future studies; the paper stands as the sequel of our previously published paper regarding the MOA of triterpenic acids with therapeutic value. The recent literature published on the topic has highlighted the role of triterpenic acids in several signaling pathways including PI3/AKT/mTOR, TNF-alpha/NF-kappa B, JNK-p38, HIF-α/AMPK, and Grb2/Sos/Ras/MAPK, which trigger their various biological activities.

## 1. Introduction

Even if the study of diagnosis, treatment, and prevention of diseases is as old as mankind itself [1] and important advances have been made throughout time, diseases have also evolved, being sustained by modern environmental factors which lead to a significant increase in the incidence of cancer, obesity, and diabetes, among other diseases [2]. Multiple drugs developed to treat these pathologies have been able to improve the lives of numerous patients, but their associated side effects, high cost, as well as the emergence of drug resistance currently limit their use [3,4]. In the search for new drugs able to overcome these limitations, phytocompounds and their derivatives have entered the scrutiny of several research groups due to their wide distribution in numerous plant species, and extensive biological spectrum proven by both in vitro and in vivo studies [5,6]. Triterpenes are well-represented phytocompounds with over 30,000 representants [7] that have received increased attention as a result of various biological properties, including anticancer, anti-inflammatory, hepatoprotective, antiviral, cardioprotective, and antimicrobial effects [8]. Among them, some triterpenic acids have been found to exert selective antiproliferative effects triggered mainly by apoptotic-dependent mechanisms [9,10,11,12]. Despite their remarkable properties, their lipophilic core imprints unfavorable physical-chemical properties that greatly hamper their bioavailability and consequent development as therapeutic drugs [12]. Thus far, to overcome these limitations, many attempts have been made which fall in the following categories: (1) chemical derivatization [13]; (2) the formation of inclusion complexes with natural or semisynthetic cyclodextrins [14]; and (3) the inclusion in nanoformulations [15], which were used either alone or in various combinations by several research groups. Studying the MOA of a drug candidate early in the development process can contribute to the early assessment of possible side effects and drug interactions that could take place inside the human organism. Currently, these experiments are mainly performed in vitro (cell cultures), ex ovo (embryonated eggs), or in vivo (small experimental animals) [16]. Although most types of experiments confirm the existence of the same mechanism of action [17], sometimes they can lead to conflicting results that could be partly explained by the complex interactions that can occur in a living organism compared with a controlled cellular environment or by the particularities of different types of cancer cell lines [18].

**Figure 1:**
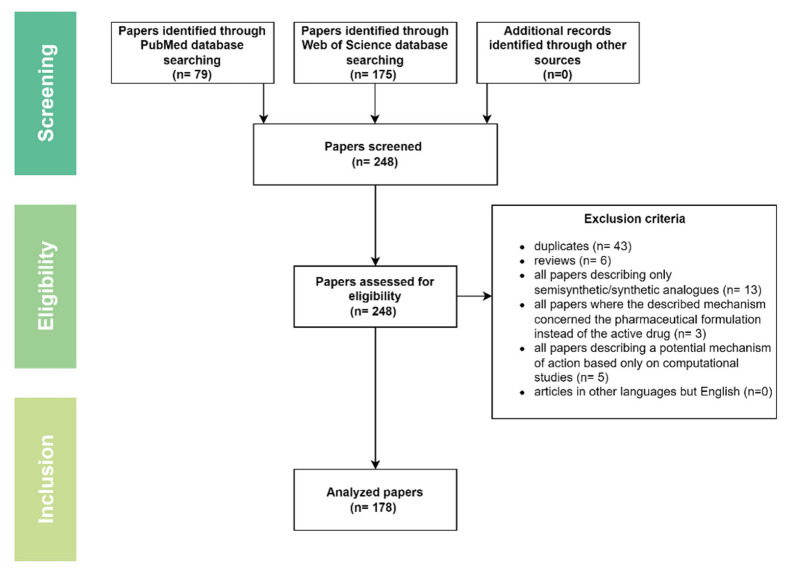
Flow diagram describing the data selection process.

This review represents the continuation of our recently published paper regarding the mechanisms of action of several triterpenic acids of therapeutic value including asiatic, oleanolic, and ursolic acid [19]. The current paper depicted the most recent (last 5 years) studies on the MOA of betulinic acid, boswellic acid, glycyrrhetinic acid, madecassic acid, moronic acid, and pomolic acid in order to provide scientists with updated and accessible material on the topic that could set in motion future original studies. Two major databases were screened, i.e., PubMed (https://pubmed.ncbi.nlm.nih.gov, accessed on 4 February 2022) and Web of Science (WOS, www.webofscience.com, accessed on 4 February 2022), using as key search terms the main triterpenic acids’ names combined with “mechanism”; following the cross-examination of results by two experienced researchers, the following papers were included: (1) in vitro studies; (2) in vivo studies; and (3) studies describing the mechanism of action of natural triterpenic acids; and the following types of papers were eliminated: (1) duplicates; (2) reviews; (3) all papers describing only semisynthetic/synthetic analogues; (4) all papers where the described mechanism concerned the pharmaceutical formulation instead of the active drug; (5) all papers describing a potential mechanism of action based only on computational evaluation; (6) articles in other languages than English (Figure 1).

## 2. Betulinic Acid

**Figure 2:**
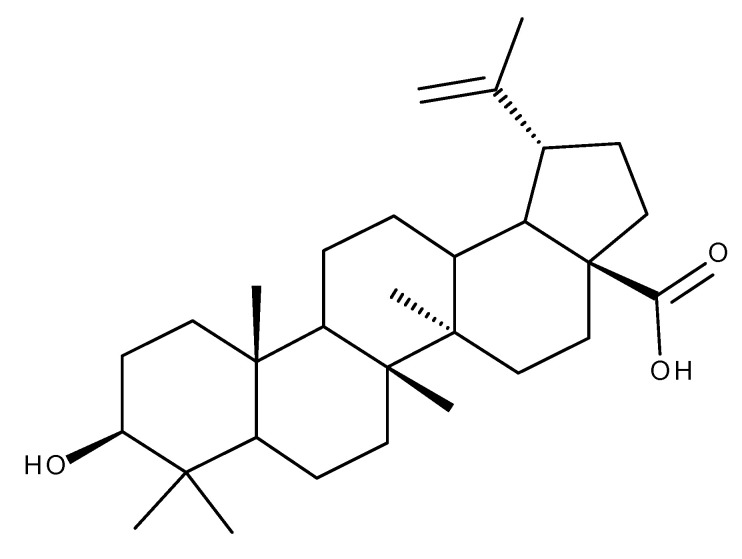
Structure of betulinic acid.

Betulinic acid (BA, Figure 2) displays a wide range of biological activities including anti-inflammatory, anti-ischemic, antimicrobial, anticancer, and antidiabetic effects. Moreover, the National Cancer Institute of the United States identified BA as one of the most promising anticancer agents currently in development, supported by numerous studies in recent years [20]. BA has also been an important scaffold for the synthesis of semisynthetic derivatives with enhanced [21] and targeted activity on the mitochondria of cancerous cells [22].

**Figure 3:**
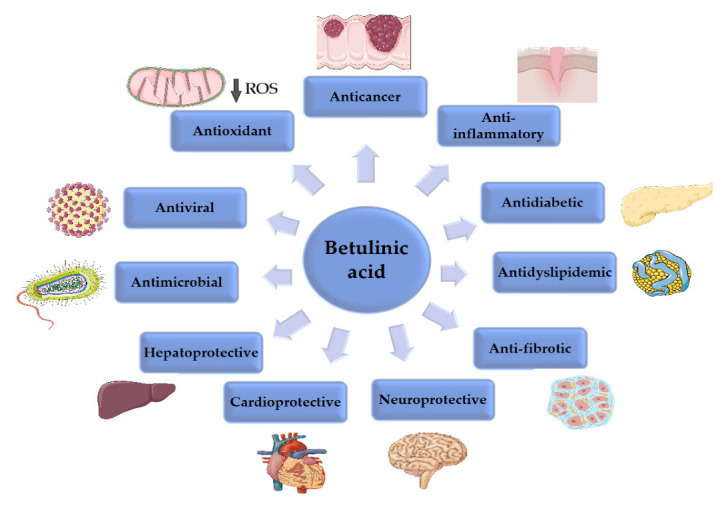
Biological activities of betulinic acid. Parts of the figure were drawn by using pictures from Servier Medical Art. Servier Medical Art by Servier is licensed under a Creative Commons Attribution 3.0 Unported License (https://creativecommons.org/licenses/by/3.0/; accessed on 2 August 2022).

A recent review by Amiri et al. offered a comprehensive insight into the previously identified mechanisms of action responsible for the plethora of biological activities possessed by BA, which are summarized in Figure 3 [23].

### 2.1. Anticancer Activity

**Figure 4:**
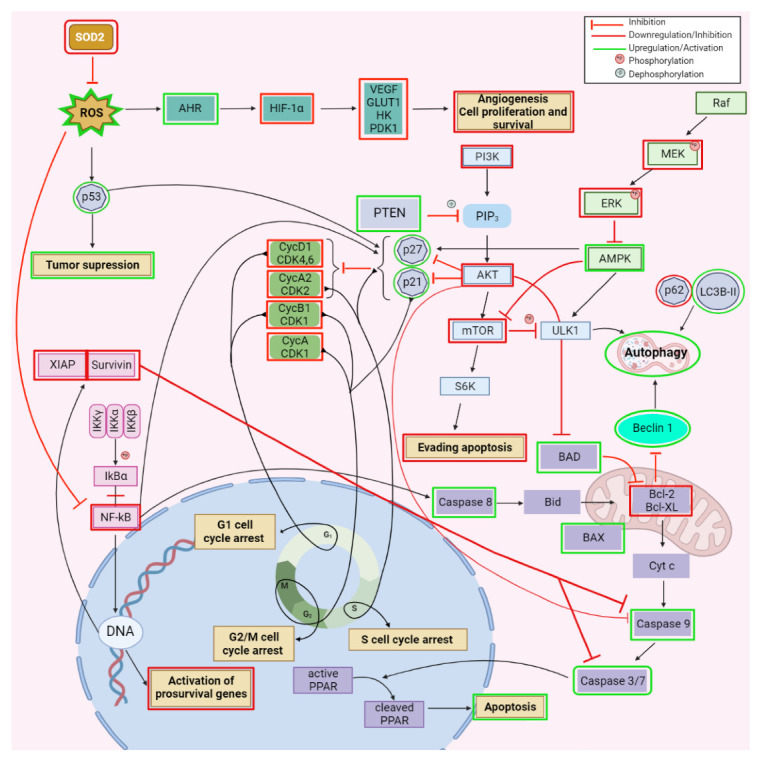
Schematic representation of the reported anticancer mechanisms of betulinic acid; key signaling pathways targeted. Created with BioRender.com (accessed on 23 June 2022).

In glioblastoma, a malignant tumor of the central nervous system, BA has shown antiproliferative effects in vitro against U87MG and A172 cell lines. The molecular mechanism suggested by the authors is the down-regulation of NF-κB pathway, which mediates survival signals in glioblastoma cells, confirmed by decreased levels of NF-κB and suppression of two factors involved in a pro-survival pathway, namely survivin and XIAP. An increase in caspase-3 and -9 was also observed, suggesting the role of mitochondria-mediated apoptosis of BA against glioblastoma (Figure 4; Table 1) [24].

In oral squamous carcinoma, in vitro as well as in vivo models showed that BA inhibited cell proliferation and decreased tumor volume through the stimulation of ROS production, which further promoted the mitochondria-mediated apoptosis, marked by the increase of caspase-3, -9, and Bax/Bcl-2 ratio. The authors concluded that the stimulation of ROS generation plays an important role in apoptosis since the effect was significantly reduced in the presence of N-acetyl-cysteine, a potent antioxidant agent [25]. Simultaneously, BA’s ability to reduce the radiotherapy resistance of CAL-27 and Tca-83, two oral squamous carcinoma cell lines, by regulating the Sp1/PTEN pathway was also revealed; BA induced the stimulation of Specificity protein 1 (SP1), which further upregulated phosphatase and tensin homolog gene (PTEN). Sp1 stimulates the expression of PTEN, whose dysfunction or down-regulation was identified in several tumors and has been associated with radiotherapy resistance; PTEN also acts as an inhibitor of the PI3K/Akt and TNF-alpha/NF-kappa B signaling pathways (Figure 4; Table 1) [26].

Complementary to these findings, BA exerted its antiproliferative effect in another drug-resistant gastric cancer cell line, SGC-7901, through the induction of autophagy as confirmed by the formation of autophagosomes and the down-regulation of ERK/MEK signaling pathway, and further confirmed by decreased levels of phosphorylated ERK and MEK proteins (Figure 4; Table 1) [27].

The antiproliferative effect of BA against hepatocarcinoma is highly selective in vitro compared with normal hepatic cells as revealed by Liu et al. who used HepG2 and SMMC-7721, two hepatocarcinoma cell lines, as well as a normal liver cell line, L-O2, for their evaluation. The study further investigated the underlying mechanism of action and identified induction of apoptosis and autophagy to be involved in the inhibition of hepatocarcinoma proliferation, mainly through the inhibition of the PI3/AKT/mTOR signaling pathway. The apoptosis induction was identified by the characteristic cell morphological changes and, at the molecular level, by increased Bax/Bcl-2 ratio and caspase-3 activity. In addition, the autophagy was confirmed by a p62 decrease and increase in LC3B-II and beclin-1 (Figure 4; Table 1). Both mechanisms were confirmed by means of 3-MA, an autophagy inhibitor, which diminished both autophagy and apoptotic markers, leading to a reduced antiproliferative effect of BA [28]. Chen et al. also confirmed the BA’s influence on autophagy and apoptosis in PLC/PRF/5 and MHCC97L hepatocarcinoma cell lines and deepened their studies in terms of molecular mechanisms; BA was found to inhibit the expression of long noncoding RNA MALAT1 (lnc-RNA Malat 1), a metastasis prognostic marker for human hepatocarcinoma, which increased the expression of miR-22-3p, further stimulated the degradation of inhibitory apoptosis proteins (IAPs), and finally triggered cell apoptosis (Table 1) [29].

The aggressiveness and apparent absence of signs and symptoms in the early stages of pancreatic cancer are responsible for the high mortality of this malignancy. The current literature suggests that BA exerts its antiproliferative effect in pancreatic cancer by the induction of mTOR-mediated apoptosis without any influence on autophagy or other apoptotic triggering pathways, such as NRf2 and JAK2, as was shown in vitro in PANC-1 and SW1990 cells and in vivo on a mouse xenograft model. Since the mTOR pathway plays a crucial role in the development and division of cells and was found to be up-regulated in various malignancies including pancreatic cancer, the inhibition produced by BA significantly diminished the in vitro proliferation of malignant cells and reduced the tumor dimension in vivo (Table 1) [30].

New treatments for acute myeloid leukemia, a severe hematological disorder, are still in high demand due to the fast emergence of drug-resistance. Previous studies have showed that BA and its derivatives presents an antileukemia effect [31,32] that could be linked to the inhibition of topoisomerases [33]. More recent studies have shown that BA is able to selectively suppress the growth of three leukemia cell lines, Kasumi-1, HL-60, and THP-1, through superoxide dismutase 2 (SOD-2) suppression and the subsequently increased ROS generation, which lead to aryl hydrocarbon receptor (AHR) activation and hypoxia-inducible factor-1α (HIF-1α) suppression. SOD-2 is an antioxidant enzyme located inside the mitochondria; following its suppression an increase in ROS occurred which subsequently up-regulated the expression of AHR, a mediator of the carcinogenetic effects of different environmental contaminants on the organism, which further inhibited the HIF1α involved in cell survival in anaerobic conditions, characteristic in cancer (Figure 4; Table 1) [34]. Complementary to these findings, in another study regarding the effect of BA against the u937 leukemia cell line, the induction of apoptosis and G2/M phase cycle arrest were identified as the underlying mechanisms of action responsible for the cytotoxic effect. The massive increase of ROS levels was responsible for both the pro-apoptotic effect as confirmed by the increased rate of Bax/Bcl-2, activation of caspase-3, -9, and PARP degradation as well as the phase cycle arrest in G2/M as confirmed by the upregulation of cyclin-dependent kinase inhibitor p21WAF1/CIP1 and decreased in cyclin A and B1. Both mechanisms were abolished when the cells were incubated with NAC, which reduced the intracellular ROS levels [35]. Another hematological disorder that could benefit from the antiproliferative effect of BA is multiple myeloma, in which BA induced S-cycle arrest and ROS mediated against apoptosis; at the molecular level, BA inhibited the NF-κB pathway followed by the overwhelming increase in ROS, which triggered the intrinsic apoptotic pathway confirmed by the downregulation of Bcl-2, upregulation of Bax, activation of caspase-3, -8, and -9, and increase in cleaved PARP and cyt C. The S cell cycle arrest was a result of two mechanisms: the inhibition of the cyclin A2/CDK2 complex and stimulation of p21^Waf1/Cip1^ and p27^Kip^ (Figure 4; Table 1) [36].

Kutkowska et al. studied the effect of BA on three non-small lung cancer cells, A549, H358, and NCI-H1703, both in hypoxic and normoxic conditions in order to simulate the tumor environment which may contain both normoxic and hypoxic regions, the latter being associated with chemotherapy resistance. BA induced cytotoxicity by several complementary mechanisms such as G1 cell cycle arrest, confirmed by the increased expression of p21 and decreased expression of cyclin D1 and B1, induction of apoptosis confirmed by an increased Bax/Bcl-2 ratio, activation of caspases-3, -7, and formation of a PARP metabolite as well as the activation of ERK (Table 1) [37]. Enhanced activity of BA was observed in hypoxic compared with the normoxic environment, therefore suggesting that BA’s effect on resistant tumors could represent a promising lead for future research.

Breast cancer, especially the triple-negative variant, is currently in high demand for new therapeutic options due to its unfavorable prognosis and aggressiveness. In a study designed to measure the ultrastructural changes of MDA-MB-231 cells in the presence of BA, the decreased expression of Bcl-2, an antiapoptotic gene was also reported. The authors have suggested that the diminished expression of Bcl-2 could be responsible for the antiproliferative effect of BA against this variant of breast cancer [38]. Complementary to these findings, Zheng et al. identified BA as an endothelial reticulum stress trigger that inhibited the anaerobic glycolysis and metastasis of MDA-MB-23 cells. At the molecular level, BA hampered the binding of PERK to the endothelial GRP78 receptors, which are overexpressed in various tumors and associated with drug resistance, apoptosis resistance, and metastatic properties; the accumulation of PERK initiated the phosphorylation of elF2α, which subsequently inhibited β-catechin and c-Myc-mediated glycolysis. Because anaerobic glycolysis is the preferred energetic pathway for the survival, proliferation, and metastasis of cancer cells, the development of potent glycolysis inhibitors could represent an attractive opportunity for future research (Table 1) [39].

BA isolated from a methanolic extract of *Cornus walteri* (Cornaceae) was identified as the main component responsible for the antiproliferative effect of the extract against A2780 ovarian cancer cells. The underlying mechanism of action was apoptosis mediated through the decrease of Bcl-2, increase of Bax and induction of caspase-3, -8, and -9. Caspase-8 is involved in the extrinsic pathway of apoptosis triggered by receptors found at the cell surface while caspase-9 is involved in the intrinsic apoptotic pathway triggered by internal factors such as DNA damage and ROS generation. Both caspase-8 and -9 can turn procaspase-3 into its active form, caspase-3, which would further catalyze specific intracellular proteins leading to apoptosis (Table 1) [40].

BA was found to be effective against cervical cancer in vitro through the induction of apoptosis in HeLa cells. One of the molecular mechanisms involved is the suppression of the PI3K/Akt signaling pathway by increasing ROS which, in turn, suppressed the phosphorylation of Thr308 and Ser473 in Akt and the downregulation of PI3K. The enhanced expression of other pro-apoptotic factors such as p21, p27, Bad and caspase-9 were also recorded in the study. Because the incubation of cells with glutathione, an antioxidant compound, resulted in the suppression of apoptosis, ROS were identified as essential elements for the BA effect against cervical cancer [41]. The role of ROS in the BA-induced apoptosis was also identified in SiHa cells, where BA increased ROS generation. Furthermore, BA blocked SiHa cell cycle in G0/G1 phase causing DNA damage and induced apoptosis in a caspase-3-independent manner. Although caspases are important enzymes involved in apoptosis due to their role in the degradation of various substrates such as PARP and BA increased caspase-3 activity in other cancer cell lines, there have also been reports of other pro-apoptotic effects of active compounds exerted independently of caspase activity, which suggest the existence of yet undiscovered mechanisms leading to apoptosis [48]. Another molecular mechanism of BA, identified in HeLa cells, is the activation of proteasomes which can produce the degradation of HIF-1α, a triggering factor for the expression of genes involved in tumorigeneses such as VEGF, GLUT1, HK, and PDK1. HIF-1α is regulated mainly by oxygen homeostasis, but other factors can also influence its expression in tumor cells such as the activation of β1, β2, and β5 proteasomes, which are able to degrade HIF-1α and subsequently inhibit the expression of tumor-promoting genes (Table 1) [42].

Although numerous studies have shown that BA pro-apoptotic effect is ROS-dependent in various types of cancer, the effect seems to be regulated in some human bladder cancer by ROS independent pathways. For example, BA induced apoptosis and cell cycle arrest in three human bladder cancer cell lines, T-24, UMUC-3, and 5637. The molecular mechanism for the cycle arrest in the G2/M phase is related to the decreasing levels of several cell cycle regulators including cyclin A, cyclin B1, cyclin-dependent kinase (CDK-2), cell division cycle 2 (CDC-2), and Cdc25c. The cells blocked in the G2/M phase further underwent apoptosis, which was confirmed by characteristic modifications, such as the up-regulation of BAX, the activation of caspase-8, -9, -3 cascade, and an increase in cleaved PARP concentrations (Figure 4). The decrease of Snail, Slug, and matrix metalloproteinase-9 (MMP-9), three factors involved in cell migration, was also observed, suggesting that BA could inhibit cell migration by a yet unexplored mechanism of action [43]. Contrary to these findings, Zhang et al. found that apoptosis induction in EJ and T24 human bladder cell lines was abolished or attenuated when an antioxidant was used, thus suggesting that BA is able to induce apoptosis in a ROS-dependent manner. They also identified the autophagy mediated by the AMK/mTOR/ULK1 pathway (Figure 4) as a mechanism involved in the antiproliferative effect of BA, as confirmed by the enhanced AMK phosphorylation combined with the decreased phosphorylation of mTOR and ULK1, alongside the increase in LC3B-II, which support the formation of autophagosomes (Table 1) [44].

Colorectal cancer remains one of the most frequent malignancies worldwide with high mortality and morbidity rates despite the use of numerous and complex treatment protocols. A sub-fraction of an n-hexane extract of *Mesua ferrea* stem bark containing BA and α-amyrin was tested against the HCT116 cell line, revealing pro-apoptotic properties. Several signaling pathway alterations were observed under the action of the extract, including the down-regulation of WNT, HIF-1α, and EGRF, the up-regulation of p53, Myc/Max, and TGF-β, and increased levels of caspase-9. The overexpression of WNT, HIF-1α, and EGRF in colorectal cancer was previously linked to the promotion of angiogenesis and proliferation of tumor cells. The down-regulation of these factors could partially explain the antitumor effect of the BA-α amyrin extract against colorectal cancer [45]. Zeng et al. focused their study exclusively on the antiproliferative effect of pure BA against colorectal carcinoma both in vitro, using the HCT116 cell line, and in vivo, on a mouse model. They identified the induction of mitochondrial apoptosis as the underlying mechanism of BA against colorectal carcinoma due to high ROS concentrations, the downregulation of Bcl-2, upregulation of BAX, and high caspase-3 activity. The study also showed that BA inhibits the migration of HCT116 cells by reducing the expression of matrix metalloproteinases (MMP) and increasing the expression of TIMP-2, a MMP inhibitor [46]. Complementary to these findings, BA was also found to inhibit the autophagy in HCT116 cancer cell line. Initially, BA increases the protein p53 expression, an autophagy marker correlated with the initiation of autophagy that would normally sustain cell survival, but subsequently stimulates the ubiquitin-mediated degradation pathway which rapidly catabolizes p53 and hampers the autophagic flux. Because several p53 variants are expressed in different cell lines such as wild-type p53 in HCT116 and mutant p53 in HT29 and SW480 and the study also found BA to be less effective against the HCT116 compared with HT29 and SW480 cell lines, a link between BA effect and different p53 variants can be suggested (Table 1) [47].

BA could also be used to prevent the accumulation of cadmium since environmental exposure to cadmium has been associated with various malignancies [49]. Fan et al. reported that BA inhibited kidney and liver carcinogenesis produced by cadmium chloride in mice by increasing the expression of Bcl-2, decreasing Bax, and inhibiting the active caspase-3 [50]. One can notice that BA acts as an apoptosis inhibitor in normal cells as opposed to its apoptotic effect in cancer cells, thus confirming its previously reported selective action on malignant cells.

### 2.2. Anti-Inflammatory Activity

BA reduced the edema in a λ-carrageen-induced paw edema in mice as a result of a combination of anti-inflammatory and antioxidant mechanisms; the anti-inflammatory effect is triggered through the downregulation of the MAPK-COX-2-PGE2 pathway and supported by the diminished expression of COX-2 and pro-inflammatory cytokines such as IL-1α, IL-1β, IL-5, IL-6, GM-CSF, KC, MCP-1, and PGE2. Moreover, BA increased GSH levels and the activity of antioxidant enzymes such as SOD, CAT, and GSH-Px in treated mice. The antioxidant effect can contribute to edema reduction by impeding the peroxidation of membrane lipids, which may aggravate the inflammatory response [51].

**Figure 5:**
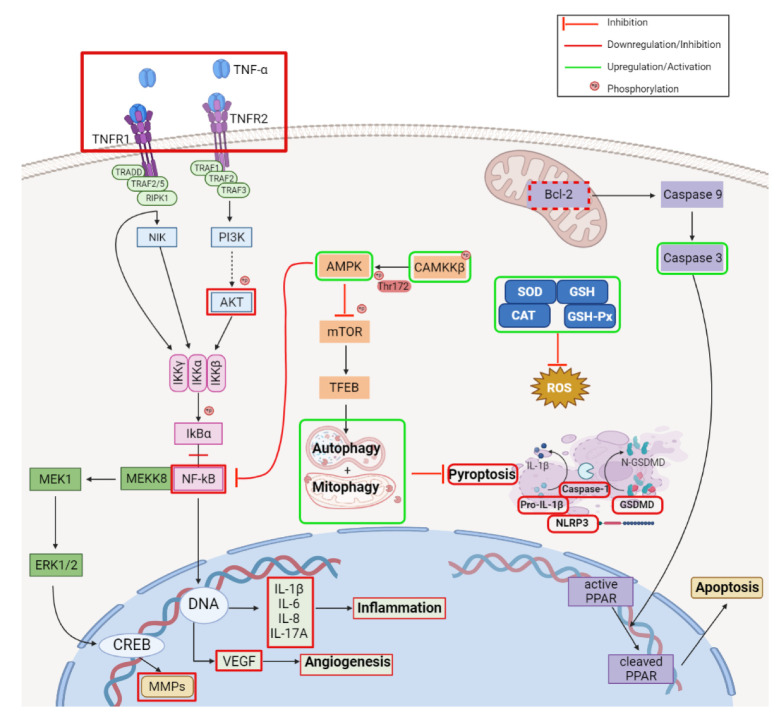
Schematic representation of the reported anti-inflammatory mechanisms of betulinic acid; key signaling pathways targeted. Created with BioRender.com (accessed on 23 June 2022).

Rheumatoid arthritis is an autoimmune disease characterized by chronic inflammation and synovial hyperplasia that can lead to cartilage destruction. BA has been found by several studies to inhibit the migration and inflammatory response of rheumatoid arthritis fibroblast-like synoviocytes (RA-FLS) both in vitro and in vivo. At the moment, the scientific literature agrees that BA’s mechanism of action in rheumatoid arthritis is linked to the inhibition of TNF-α, which further downregulates the Akt/NF-κB pathway, decreases MMPs expression, and reduces the level of pro-inflammatory cytokines including IL-1β, IL-6, IL-8, and IL-17A as well as several growth factors such as VEGF and TGF-β (Figure 5) [52,53,54].

Acute pancreatitis is a disease characterized by severe inflammatory response and the release of pancreatic enzymes into the blood that can lead to severe complications, such as multiple organ failure. BA exerted both prophylactic and therapeutic effects in acute pancreatitis in vivo by the downregulation of the NF-κB pathway, which further reduces the synthesis of proinflammatory cytokines including IL-1β and IL-6 (Figure 5); moreover, BA reduced the levels of pancreatic enzymes such as amylase and lipase, thus attenuating the progression and severity of the disease [55].

Psoriasis is an immune-mediated skin disorder characterized by inflammation and hyperplasia of the skin; nowadays, treatment includes immunosuppressive medications such as methotrexate and cyclosporine, which are associated with severe side effects. The use of BA in the management of psoriasis could be beneficial as shown by an in vivo study on mice in which BA greatly improved skin lesions in a dose-dependent manner. The underlying mechanisms consist of the inhibition of pro-inflammatory cytokine IL-17A, the increase of anti-inflammatory IL-10 cytokine, and the inhibition of the NF-κB pathway, which further inhibits the production of IL-17A, IL-6, and RORγT (Figure 5) [56].

The anti-inflammatory effect of BA was also observed in microglia cells; microglia is an immunological cell in the central nervous system associated with several neurodegenerative diseases such as multiple sclerosis, Alzheimer’s disease, and Parkinson’s disease. These cells have the ability to exhibit the M1 pro-inflammatory or M2 anti-inflammatory phenotype depending on the microenvironment conditions, the two phenotypes being distinguished by the particular pro-inflammatory and anti-inflammatory proteins they express. BA prevented the in vitro and in vivo transformation of microglia cells into the M1 phenotype stimulated by LPS, an agonist of the Toll-like receptor 4 which increases the M1/M2 ratio, thus promoting inflammation. The molecular mechanism could be related to the activation of the AMPK pathway, which has been previously proven to promote the M2 anti-inflammatory phenotype as suggested by the increased phosphorylation of CaMKKβ, a kinase that can further phosphorylate the Thr172 of AMPK and activate the pathway (Figure 5) [57]. Using the same BV-2 cell line, another study showed BA to induce apoptosis as confirmed by the decrease of Bcl-2, activation of caspase-3, and the subsequent degradation of PARP by caspase-3. Additionally, it was noticed that BA increased the conversion of LC3-I to LC3-II as well as the accumulation of p62, thus blocking the autophagic flux; however, the exact mechanism involved has not been yet identified [58].

The anti-inflammatory effect of BA in the central nervous system through the RIP140 inactivation, a cofactor of NF-κB, produced the decrease in pro-inflammatory cytokines and reduced mild-depression in mice. The authors suggested that the BA antidepressant effect could partially be a result of this mechanism [59]; however, further studies are needed to exhaustively elucidate all involved mechanisms.

Inflammation may trigger pyroptosis, a cell death mechanism which has been previously associated only with infectious diseases; more recently, pyroptosis’s role has been extended to other pathologies, including cancer [60], cardiovascular diseases [61], and spinal cord injuries [62]. Wu et al. studied the underlying mechanism of BA in spinal cord injury in mice and identified several mechanisms that might activate each other to provide alleviation in spinal cord injuries. Through the activation of the AMPK-mTOR-TFEB pathway, BA activates autophagy and augments mitophagy, which further inhibit pyroptosis, as confirmed by the decrease in pyroptotic markers such as NLRP3, caspase-1, GSDMD, and IL-1β (Figure 5) [63].

### 2.3. Antidiabetic Activity

The use of BA in the treatment of diabetes could be beneficial due to its anti-hyperglycemic effect, but its mechanism of action is not yet elucidated. However, some in vitro and in vivo studies revealed potential targets and mechanisms responsible for this effect, such as the inhibition of α-glucosidase [64]. BA can also stimulate the pancreatic insulin secretion by inhibiting the ATP-dependent potassium channels, producing the depolarization of the membrane and subsequently opening the L-type voltage-dependent calcium channels (L-VDDCC), leading to an abrupt increase in calcium concentration inside the cells associated with insulin release [65]. In support of the insulin secretagogue effect of BA, Khataylou et al. reported the increase in peptide C serum concentration in mice, a byproduct of the conversion of proinsulin to insulin. Contrary to these findings, Birgani et al. did not record an increase in insulin secretion in the tested animals [66]. These opposite findings could be a result of the different experimental methods used, since Gomes Castro et al. employed an ex vivo method, using pancreatic cells isolated from euglycemic rats while Birgani et al. administered BA parenterally to mice and decelerated the insulin concentration from the collected serum. It is also possible that the small in vivo variations of insulin concentration are difficult to assess and the determination of peptide C is a better indicator for pancreatic insulin secretion. Complementary to these studies, Song et al. suggested that in diabetes BA acts at the molecular level in the same manner as metformin, a well-established anti-hyperglycemic drug, and stimulates the AMPK pathway in a PI3K independent manner. Moreover, BA increases the expression of glucose transporter 4, which favors the intracellular glucose uptake [67].

### 2.4. Cardioprotective Activity

Oxidative stress has been associated with the pathogenesis of various cardiac pathologies and conditions that favor the formation of ROS and lead to cardiac dysfunction. Bacterial infections, especially those with gram-negative bacteria may, result in sepsis due to a membrane lipopolysaccharide (LPS) that triggers ROS generation and systemic inflammatory response. Pretreatment with BA activated the Nrf-2 pathway that subsequently triggered the transcription and synthesis of antioxidative enzymes such as SOD, HO-1, and glutathione peroxidase (GPx), which, in turn, could impede LPS-induced cardiac damage [68]. The inhibitory effect of BA on the JNK-p38 pathway has also been associated with a protective effect against dexamethasone-induced damage in lymphatic organs in mice [69], suggesting that this pathway could be a multifunctional target for the phytocompound. Moreover, BA can prevent cardiac fibrosis, characterized by the increased proliferation of cardiac fibroblasts and accumulation of extracellular matrix proteins, which can further contribute to the pathogenesis of myocardial infarction. At the molecular level, BA inhibited the glucose-induced expression of extracellular matrix proteins such as α-SMA, collagen, and fibronectin through the inhibition of the TGF-β1/Smad signaling pathway [70].

Ischemia is a pathology in which the tissue blood supply is restricted for a period of time, creating a hypoxic environment that favors the anaerobic metabolism, ROS generation, and cell death by necrosis, apoptosis, or autophagy; it is associated with widespread pathologies such as myocardial infarction and ischemic stroke. The ischemic episode is followed by the reperfusion of the ischemic tissue, which may, however, aggravate the injury through pro-inflammatory cytokine infiltration [71].

After an ischemic stroke episode, the expression of enzymes involved in ROS generation is increased. Prophylactic treatment with BA was found to inhibit the expression of enzymes such as NADPH oxidase 4 (NOX4), an enzyme largely distributed in the blood vessels, and to diminish ROS formation [72]. The activation of pro-inflammatory and autophagy pathways can also be noticed after an ischemic stroke; BA downregulated the HIF-α/AMPK proinflammatory pathway [73] and activated SIRT/FoxO1 responsible for the inhibition of autophagy [74].

In myocardial infarction, BA exerts its anti-ischemic properties by reducing the hypoxia effects produced during the ischemic episode, such as ROS generation and the subsequent initiation of apoptosis; the molecular mechanisms involved are the activation of the antioxidant Nrf2-hemo oxygenase-1 and the downregulation of JNK-p38 pro-apoptotic pathway [75].

Atherosclerosis is a chronic disease with an insidious debut, involved in the pathogenesis of hypertension, diabetes, and hypercholesterolemia, pathologies associated with high morbidity rates. Endothelial dysfunction has been identified as an early marker for the initiation of atherosclerosis; thus, the use of compounds that ameliorate the endothelial dysfunction could hamper atherosclerosis progress and prevent the occurrence of associated diseases. As such, BA was found to reduce endothelial dysfunction by increasing the expression of endothelial nitric oxide synthase (eNOS), which further inhibited the expression of intracellular adhesion molecule-1 (ICAM-1) and endothelin-1 (ET-1) that promote the atherosclerotic plaque formation [76].

### 2.5. Lipolytic Activity

Obesity is a major health problem characterized by the imbalance of the lipogenesis–lipolysis equilibrium caused by multiple factors such as poor diet, stress, genetic predisposition, lifestyle, and other associated comorbidities. Anti-obesity drugs that can restore lipid balance by stimulating lipolysis and/or inhibiting lipogenesis and adipocyte formation are in high demand to treat the now so-called obesity epidemic. In vivo, BA can interfere in the differentiation process of human mesenchymal stem cells which can undergo either osteogenesis or adipogenesis; BA upregulates UCP-1 and PGC-1α and downregulates C/EBP- α, mechanisms that inhibit adipogenesis and promote osteogenesis [77]. In addition, BA is able to inhibit adipocyte differentiation and stimulate lipolysis through the activation of the AMPK pathway that in turn regulates the expression of key enzymes within the lipid metabolism alongside the downregulation of PPARγ [78]. The expression of PPARγ, a key nuclear transcription factor involved in several metabolic pathways including lipid metabolism and thus an important target for drug development, can be suppressed by BA but the underlying mechanism has not yet been fully elucidated [79]. Current studies suggest that the downregulation of PPARγ by BA is a result of the inhibition of PI3K/AKT signaling pathway [80] and/or the modulation of the interaction between PPARγ and its co-activators [81].

There is a strong association between obesity and nonalcoholic fatty liver disease (NALFD), with 70% of obese individuals suffering from a form of NALFD. BA has been found to promote fatty acid oxidation and diminish the endoplasmic reticulum stress, lipogenesis, fibrosis, and inflammation in NALFD both in vitro and in vivo. In terms of the molecular mechanism, BA alleviated the endoplasmic reticulum stress through a double mechanism: the stimulation of FXR and inhibition of PERK/EIF2α signaling pathways, which promote fatty acids oxidation (Gu 2018, DOI: 10.1111/bph.14570). BA can also delay the accumulation of triglycerides in hepatocytes through the inhibition of fatty acid synthase by the upstream transcriptional suppression of Ying Yang 1 (YY1) [82].

### 2.6. Antibacterial and Antiparasitic Activity

The treatment of bacterial infections remains an important application of phytocompounds due to the rapidly evolving bacteria that become resistant to current treatments. BA, as well as other triterpenic compounds, exerts its antibacterial effect against various strains through a ROS-dependent mechanism. In support of this proposed mechanism, Oloyede et al. found that BA enhances ROS production by stimulating the electron transport chain, as confirmed by the increase of the NAD+/NAD ratio in *Escherichia coli*, *Pseudomonas aeruginosa*, and *Staphylococcus aureus* strains [83].

ROS increase by BA is also responsible for the antiparasitic effect against *Trypanosoma cruzi*, which causes Chagas’ disease; BA presented superior selectivity to the currently used benznidazole against all *Trypanosoma cruzi* stages of development [84].

### 2.7. Effect against T2-Mycotoxin Induced Toxicity

T-2 is an A-trichothecene mycotoxin contaminant found in various agricultural products that can lead to multiple organ dysfunctions in humans. It is unanimously accepted that T-2 exerts its toxic effect by a mechanism that greatly increases oxidative stress, which later activates mitochondrial-mediated apoptosis in healthy cells. BA pretreatment in mice offered protection against T-2 induced testicular injury through the inhibition of the JAK2/STAT3 pathway, which led to the increase of antioxidant capacity and the decrease of pro-apoptotic mediators such as Bax and caspase-3 [85].

Zhu et al. found that BA exerts protective effects against T-2 induced cytotoxicity in the thymus through the inhibition of MAPK and activation of Nrf2 signaling pathways, which synergistically reduced oxidative stress by both diminishing the ROS production and increasing the antioxidant enzymes such as HO-1 [86]. This mechanism was also confirmed by Kong et al. in a study conducted to elucidate the BA mechanism in T-2-induced spleen toxicity where the authors reported similar results [87]. Further studies are needed to determine if this mechanism also contributes to the BA’s effect in other organs in which T-2 toxicity has been reported.

Since the ingestion of contaminated products is the main entry for T-2 toxin in the organism, intestinal dysfunctions are frequently associated with T-2 toxicity. Luo et al. found that pretreatment with BA ameliorated the intestinal inflammation in mice through the inhibition of the NF-κB signaling pathway, which increased the anti-inflammatory/ pro-inflammatory cytokine ratio [88]. BA pretreatment also diminished intestinal injury produced by cyclophosphamide through the same NF-κB pathway inhibition [89], suggesting that this pathway could represent a valuable target for the development of new molecules able to treat intestinal dysfunctions.

### 2.8. Other Biological Activities

BA was identified as the main anti-tyrosinase phytoconstituent of *Dillenia indica* extract, frequently used as a food additive due to its antioxidant properties; BA exerts its effect through a non-competitive enzymatic inhibition which leads to the alteration of the tyrosinase catalytic site, resulting in the diminishing of the oxidative reactions mediated by the enzyme [90].

Kaundal et al. studied the protective effect of BA in streptozotocin-induced cognitive impairment in rats; the authors revealed multiple underlying mechanisms. BA treatment inhibited acetylcholinesterase, reduced the oxidative stress, and increased neurotransmitter concentration in the hippocampal area, which overall improved behavioral test results in treated rats [91].

In nephropathy, BA isolated from *Syzygium cumini* reduced the associated in vivo proteinuria through the downregulation of the NF-κB pro-inflammatory pathway and upregulation of the Nrf2 antioxidant pathway, as confirmed by the reduced expressions of pro-inflammatory cytokines TNF-α and iNOS and increased expression of two antioxidant factors, HO-1 and NQO1 [92]. Moreover, BA exerts an anti-fibrotic effect in renal fibrosis by the inhibition of TGF-β, which has been shown to inhibit fibroblast proliferation alongside its previously reported NF-κB inhibitory effect [93].

Administration of BA prevented the postoperative necrosis of skin flaps in vivo through the stimulation of neovascularization and autophagy and inhibition of oxidative stress, which subsequently inhibited the apoptosis of skin cells. Although the complete mechanism has not yet been discovered, the study revealed the role of some key factors such as VEGF and cadherin 5 into the BA-induced neovascularization [94].

Endometriosis is a gynecological inflammatory disease that affects between 5% and 10% of women during the reproductive phase; in endometriosis, an increase in the overexpression of estrogen receptor β (ER-β) compared with estrogen receptor α (ER-α) has been reported, which promotes endometriotic cell proliferation and inflammation. BA was found to suppress ER-β expression by targeting ER-β promoter genes that further induced apoptosis in endometriotic cells and decreased the production of pro-inflammatory cytokines including IL-1β, IL-6, and TNF-α [95].

## 3. Boswellic Acid

**Figure 6:**
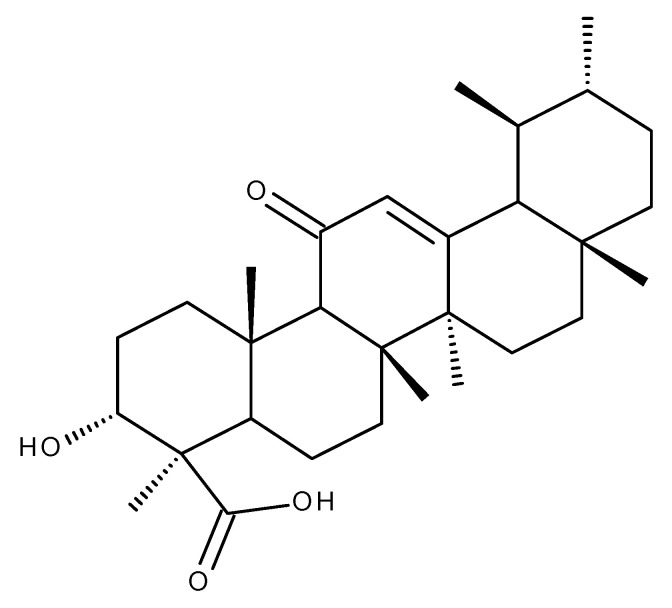
Structure of boswellic acid.

Boswellic acid (BoA, Figure 6) is a pentacyclic triterpene obtained from the gum resin of *Boswellia* sp. also known as Olibanum or Frankincense. 

**Figure 7:**
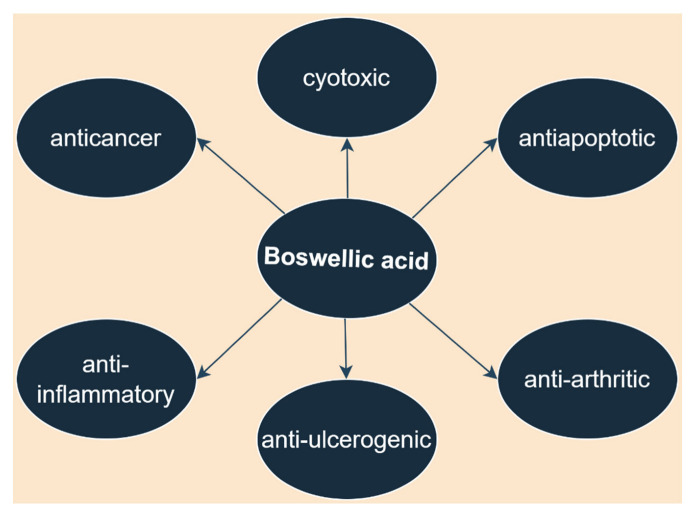
Biological activities of boswellic acid. Parts of the figure were drawn by using pictures from Servier Medical Art. Servier Medical Art by Servier is licensed under a Creative Commons Attribution 3.0 Unported License (https://creativecommons.org/licenses/by/3.0/, accessed on 2 August 2022).

As demonstrated by numerous studies and pinpointed in a recent review, BoA possesses numerous biological activities such as anti-inflammatory, anti-ulcerogenic, anti-arthritic, antiapoptotic, cytotoxic, and anticancer activities (Figure 7) [96]. However, the underlying molecular mechanisms responsible for its extensive biological activities are still poorly understood.

### 3.1. Anti-Inflammatory Activity

**Figure 8:**
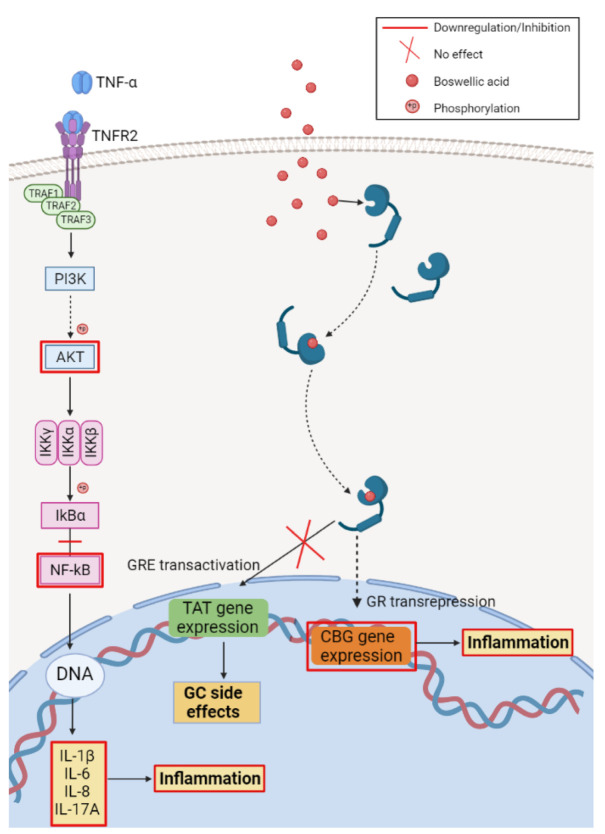
Schematic representation of the reported anti-inflammatory mechanisms of boswellic acid; key signaling pathways targeted. Created with BioRender.com (accessed on 23 June 2022).

In terms of anti-inflammatory molecular mechanisms, the recent work of Zang et al. described the interaction between BoA and the glucocorticoid receptor (GR). By employing fluorescence polarization competitive binding assay and immunofluorescence assay, the group reported a dose-dependent binding of BoA to the GR in HeLa human cervical cancer cells followed by the nuclear translocation of the resulting BoA-GR complex from the cytoplasm. However, further analysis assessing the capacity of BoA to mediate GR transcriptional activity showed that BoA does not induce the glucocorticoid response element (GRE)-mediated transcription. Furthermore, in macrophage-like U937 cells, BoA inhibited the production of pro-inflammatory cytokines TNF-α and IL-1β (Figure 8).

In HepG2 hepatocellular carcinoma cells, BoA down-regulated the corticosteroid-binding globulin (CBG) gene expression, a marker for GR transrepression, without up-regulating tyrosine aminotransferase (TAT) gene expression, a marker of GRE transactivation (Figure 7). By means of molecular docking, the same group showed that α-boswellic acid-GR binding is stabilized by hydrogen-bonding as well as hydrophobic interactions. These results indicate BoA’s dissociated activities, in particular its ability to separate transrepression from transactivation. Since recent clinical studies found that the side effects produced by glucocorticoids are related to the GRE transactivation, the authors proposed that BoA could be a selective GR modulator against inflammatory disorders (Figure 8) [97].

### 3.2. Antidiabetic Activity

The antidiabetic activity and the possible underlying mechanisms of BoA were investigated in vivo on a high-fat diet and low dose streptozotocin-induced type 2 diabetes animal model; the authors reported a significant reduction in blood glucose levels together with a decrease of total cholesterol (TC), triacylglycerol (TG), and low-density lipoprotein cholesterol (LDL-C) [98]. Considering the increasing number of studies that bring evidence to the involvement of oxidative stress in diabetes, and more specifically in impaired glucose tolerance, insulin resistance, beta-cell dysfunction, and diabetic complication [99], the antioxidant effect of BoA was also evaluated. The results showed that the diabetic animals treated with BoA exhibited decreased malondialdehyde (MDA) and increased superoxide dismutase (SOD) serum levels compared with the control group, thus indicating the antioxidant effect of the compound. Moreover, docking studies revealed the binding preference of BoA towards dipeptidyl peptidase 4 (DPP-4), a glycoprotein involved in glucose metabolism, apoptosis, immune regulation, and degradation of Glucagon-like peptide-1 (GLP-1); the inhibitory ability of BoA against DPP-4 and GLP1 was validated by in vitro testing [98].

Similar results on MDA levels were described in a wound healing experimental model of diabetic animals; the BoA treatment significantly inhibited the streptozotocin-increased MDA levels, decreased serum glucose and glycated Hb levels, and increased serum insulin levels. Additionally, BoA downregulated the proinflammatory cytokines TNF-α, IL-1β, IL-6, and NF-κB and the pro-apoptotic Bax mRNA expressions, thus revealing its implication in inflammation, oxidative stress, and apoptosis. The study revealed that the delayed wound healing observed in diabetes seems to be associated with an increased inflammatory response in the wound area and may also be positively correlated with an increase in Bax expression/apoptosis rate, responsible for the decrease of fibroblast proliferation and collagen synthesis [100].

Moreover, the same study revealed that BoA significantly upregulates Ang-1, Tie2, VEGF, and collagen-1 mRNA expressions [100]. VEGF is a crucial angiogenic factor and its activity is modulated by Ang-1 and consecutively by Tie-2, a potent agonist of Ang-1 involved in maintaining the vascular stability [101]. Since diabetes induces a defective angiogenic response responsible for the delayed wound healing [102], BoA interaction with Ang-1/Tie-2, VEGF, and collagen-1 suggests that BoA promotes angiogenesis, increases collagen synthesis, and ultimately accelerates wound healing in diabetic models [100].

### 3.3. Anti-Infectious Activity

The recent study of Goswami et al. [103] reported the antiviral effect of BoA against Herpes simplex virus-1 (HSV-1) in African green monkey kidney Vero cells at an early stage of virus replication (1–4 h post-infection). Infection with HSV-1 triggers a cascade of intracellular signaling events involving: (i) NF-κB activation and consecutive TNF-α and IL-6 pro-inflammatory cytokines release [104] and (ii) p38 MAP-kinase activation that promotes viral replication [105]. In terms of molecular mechanisms, BoA produces an anti-inflammatory effect by downregulating the virus-induced TNF-α, IL-1β, and IL-6 production. Furthermore, in HSV-1-infected peritoneal macrophages, the authors also reported the downregulation of NF-κB and p38 MAP-kinase activation after BoA treatment, therefore leading to the reduced expression of pro-inflammatory genes and fast control of virus spreading [103].

### 3.4. Neuroprotective Activity

The literature review shows that BoA has an immense potential in nervous system disorders treatment [106]. As recently demonstrated in streptozotocin-induced sporadic Alzheimer’s disease in rats, BoA produced an anti-neurodegenerative effect by increasing the expression of reelin and subsequently decreasing tau protein hyperphosphorylation [107]. Reelin, a large extracellular glycoprotein involved in the modulation of synaptic neurotransmission, neuronal plasticity, and memory, triggers a kinase cascade involving the activation of Dab1 and then PI3K and Akt, which leads to the inhibition of GSK3β and finally to the reduction of tau phosphorylation [108,109]. Moreover, in the same experimental model, BoA significantly improved learning and memory abilities of treated rats [107]. Another study aimed to investigate the effects of BoA in rats with trimethyltin-impaired learning and memory capacity and found that BoA has a neuroprotective effect and improves cognitive function; the underlying mechanisms of action are AChE inhibition and antioxidant activity, revealed by its capacity to decrease MDA and increase GHS levels [110]. Similar results were obtained by Afzal et al. [111] in rats with scopolamine-induced dementia and neuronal damage; the authors reported that BoA treatment significantly improved spatial learning abilities, reversed behavioral changes and memory loss, and decreased the oxidative stress in experimental animals. Specifically, BoA increased the antioxidant enzymes GSH, CAT, and SOD and decreased MDA levels while simultaneously attenuating the scopolamine-induced increased level of AChE. Moreover, BoA reestablished the tmRNA expressions of cAMP-response element-binding (CREB), Brain-Derived Neurotrophic Factor (BDNF), Cyclic adenosine monophosphate (cAMP), Ca2+/calmodulin-dependent protein kinase (CaMK), and ERK, and PI3K initially decreased after scopolamine treatment.

### 3.5. Other Biological Activities

As reported by various studies, inflammation and oxidative stress play a key role in tissue damage and are strongly intercorrelated with regard to the negative effects after exposure to environmental pollutants, bisphenol-A (BPA) and γ-radiation (IR) [112]. One study that followed these effects found that both pollutants produced cardio-/hepatotoxicity in animal models, while BoA exhibited a protective effect through its anti-inflammatory and antioxidant properties. BoA decreased the damage caused by ROS-induced inflammatory pathways via modulation of hepatic peroxisome proliferator activated receptors-alpha PPAR-α/P38 and cardiac calcineurin-A/NFATc1/P38 pathways, while lowering the systemic inflammatory mediators’, IL-6 and TNF-α, serum levels [113]. Specifically, p38 mitogen-activated protein kinases (MAPKs) up-regulates PPAR-α, a transcription factor involved in liver lipid metabolism as well as in the hepatic inflammatory response that can induce systemic inflammation [114]; BoA treatment enhances the expression of the hepatic PPAR-α/p38 signaling axis and consecutively ameliorates the hepatotoxicity induced by BPA and IR exposure (10.1080/13813455.2020.1727526). In BPA and irradiated animals, BoA suppressed cardiac hypertrophy and remodeling by decreasing the ET-1/IP3 mediated activation of the calcineurin A/NFATc1 signaling axis [113]

## 4. Corosolic Acid

**Figure 9:**
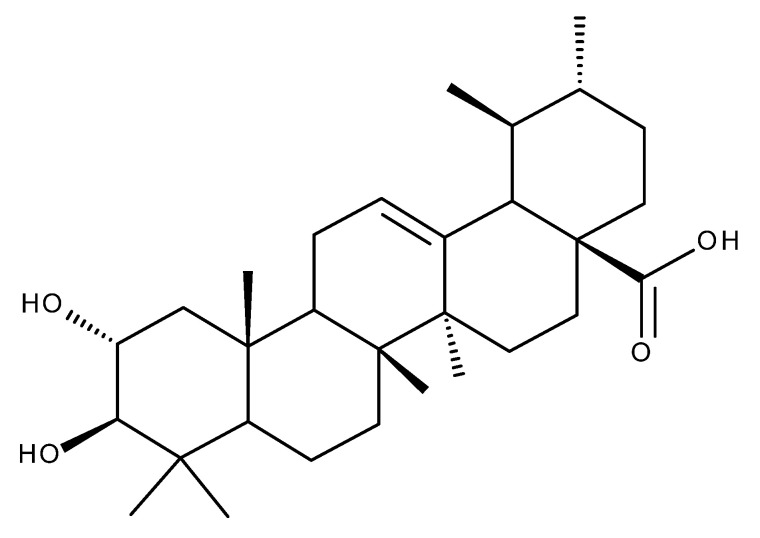
Structure of corosolic acid.

A large number of studies have reported the beneficial antidiabetic, anti-inflammatory, and anti-tumor effects of corosolic acid (CA, Figure 9), a pentacyclic triterpenoid extracted mainly from banaba leaves (*Lagerstroemia speciosa*) and loquat (*Eriobotrya japonica*) [115].

### 4.1. Anticancer Activity

Corosolic acid has revealed anti-tumor effects in various types of cancer such as colorectal, gastric, renal, liver, lung, brain, and prostate.

In order to improve the poor prognosis of gastric cancer, the world-wide second leading cause of cancer-related deaths, the current research focused on overcoming the most difficult obstacle in cancer therapy: drug-resistance. In this context, CA was revealed to be a useful option in decreasing gastric cancer chemoresistance to 5-fluorouracil, the most frequently prescribed chemotherapeutic agent for this type of cancer. Park et al. demonstrated that there are differences between 5-fluorouracil-resistant and -sensitive gastric cancer cells in terms of AMPK pathway phosphorylation level; by activating the AMPK pathway, CA restored the chemosensitivity of 5-fluorouracil-resistant gastric cancer cells [116]. Another study on human gastric cancer reported that CA produces its pro-apoptotic effects via the inhibition of NF-kB (p65) nuclear translocation. Furthermore, CA up-regulated the mRNA and protein expression of Bax and IκB-α, which inhibit the NF-κB transcription factor, and down-regulated the protein expression of p65, p-IκB-α, Fas, smac, and Bcl-2; the crosstalk between NF-kB and apoptosis-related proteins (survivin, Fas, Bcl-2/Bax, smac, caspase-3, etc.) or other protein regulators was suggested by the authors as the missing link between CA and NF-kB suppression (Table 2) [117].

In colorectal cancer which is the third-most common cancer worldwide, CA produced its anti-cancer effects by inhibiting the neuregulin1 (NRG1)-induced heterodimerization of HER2 and HER3 and their consecutive phosphorylation [118]. HER2 and HER3 are two members of the human epidermal growth factor (ErbB) receptor family that, upon their activation by the NRG1 ligand, play a significant role in cancer cell proliferation, differentiation, and migration. In patients with colorectal cancer, HER2 and HER3 tumor levels are positively correlated with reduced survival rates [119]. Moreover, as reported by the same research group, CA antitumor mechanism is not limited to HER2/HER3 pathway inhibition; the results of in vitro and in vivo colon carcinoma xenograft models revealed that, by down-regulating HER2/HER3, CA also inhibited its two classic down-stream pathways PI3K/Akt and Ras/Raf/MAPK, thus decreasing the Drp1 phosphorylation, which resulted in mitochondrial dynamics changes responsible for the CA anticancer effects (Table 2) [118].

Positive results after CA treatment were also obtained in diabetes-associated liver cancer. Studies found a significant increase of YAP levels in liver cancer and identified it as a potent oncogene [126] whose O-GlcNAcylation by O-GlcNAc transferase regulates cancer cell growth and tumorigenesis [127]. In various liver cancer cell lines exposed to high glucose conditions, CA inhibited the activity of cyclin-dependent kinase 19 (CDK19) that in turn reduced the Yes-associated protein (YAP)-O-GlcNAcylation pathway and thus decreased cancer cell proliferation and tumor growth [120]. Jia et al. [121] also reported the involvement of YAP pathway in CA mechanism of action in Hep3B and HepG2 hepatocellular carcinoma cell lines; upon CA treatment, YAP was translocated from the nucleus to the cytoplasm and its binding to CREB, Runx2 and TEAD transcription factors was reduced (Table 2). 

The literature shows that ROS are key players in promoting cancer cell proliferation [128]; however, they are regarded as a double-edge sword, other numerous studies reporting that increased ROS production can cause an opposite effect, leading to the induction of cancer apoptosis [129]. In vitro studies on ACHN and A498 renal cancer cell lines revealed another mechanism for CA as anticancer agent; it appears that CA treatment significantly increased ROS production and induced a non-apoptotic, non-necroptotic, and non-ferroptotic cell death that was alleviated by α-tocopherol, a lipophilic antioxidant, thus suggesting the mechanism behind CA activity is the increase of lipid peroxidation [122].

CA was also able to inhibit cell growth and induce apoptosis in castration-resistant prostate cancer; the underlying mechanism of CA-induced cell survival inhibition involves the increase of binding immunoglobulin protein (Bip) expression, a marker protein of endoplasmic reticulum (ER) stress [123]. Normally, ER is involved in protein folding and maturation; however, increased demand or folding aberrations will lead to the accumulation of misfolded protein, also known as ER stress; as a result, a cell-signaling system aimed to restore protein homeostasis is activated (the unfolded protein response, UPR) [130]. Through Bip activation, CA activates the ET stress and its associated two pro-apoptotic pathways: IRE-1/ASK1/JNK and PERK/eIF2α/ATF4/CHOP. The downstream effector of the IRE-1/ASK1/JNK pathway is the mitochondria where CA finally induced the intrinsic mitochondria-dependent apoptotic pathway. The PERK/eIF2α/ATF4/CHOP pathway activated in cascade by CA up-regulated the ER stress-inducible gene TRIB3 expression that consecutively inhibited p-AKT and increased mitochondrial Bax expression; these events finally led to a decreased cell survival and cancer progression (Table 2) [123].

Studies reported that in brain cancers the abnormal activation of the receptor tyrosine kinase family is correlated with uncontrolled proliferation, apoptosis inhibition, and metastatic progression [131]. In glioblastoma, the overexpression of AXL kinase, a member of the receptor tyrosine kinase family, and AXL ligand growth arrest-specific 6 (Gas6) was correlated with a poor patient prognosis [132]. An in vitro study of four glioblastoma cell lines revealed that CA’s anti-metastatic mechanism is a result of AXL pathway down-regulation and, more precisely, CA stimulates the polyubiquitination/proteasomal degradation of AXL and subsequently inhibits the JAK2/MEK/ERK axis. Continuing the investigations on the CA link to AXL, the same paper reported that the upstream regulator of AXL, carboxyl terminus of Hsc70-interacting protein (CHIP), is also upregulated after CA treatment (Table 2) [124].

Using an in vitro model of human retinoblastoma, Wang et al. [125] reported that the disruption of the maternal embryonic leucine-zipper kinase (MELK)-FoxM1 signaling pathway is the mechanism behind CA cytotoxic and pro-apoptotic effects. FoxM1 is a transcription factor involved in cancer growth, development, and progression [133], whereas its activity is regulated by MELK, a serine/threonine kinase. Treatment of Y-79 human retinoblastoma cell line with CA was able to inhibit the expression levels of MELK and FoxM1 while also reducing the transcriptional activity of FoxM1 to a baseline level (Table 2) [125].

### 4.2. Antidiabetic Activity

Increased literature evidence showing the strong antidiabetic activity labeled CA as the “plant insulin”; however, there are few recent papers which intended to elucidate the mechanism that endorses this effect.

Phosphoenolpyruvate carboxykinase (PEPCK) is an enzyme found in the liver and kidney where it catalyzes the transformation of oxaloacetate to phosphoenolpyruvate, one step in the gluconeogenesis mechanism. Since PEPCK overexpression has been described in all diabetes models, it can be used as an indicator of blood glucose [134]. In HepG2 cells and in diabetes rat and zebrafish models, CA treatment increased glucose consumption and stimulated glycogen accumulation by inhibiting mRNA expression of PEPCK [135]. Interestingly, CA also proved to inhibit the expression of GLUT1, GLUT2, GLUT3, LDHA, and LDHB and decreased the expression of GP, GYS1, G6Pase, PFKFB3, and INSR, key enzymes in carbon metabolism, thus suggesting the enhancement of glycolysis [135]. Improvement in insulin sensitivity was also reported by another study after CA treatment; this beneficial effect was demonstrated to be a consequence of AMPK activation via LKB1, an upstream kinase that phosphorylates the α-kinase subunit at the Thr172 residue of AMPK, thus leading to AMPK activation.

### 4.3. Cardioprotective Activity

Due to its plethora of beneficial activities, research groups were also interested in the potential cardioprotective activity of CA and its possible mechanism of action. CA reduced cardiac dysfunction and fibrosis during cardiac remodeling following an acute myocardial infarction by modulating the oxidative stress, inflammatory, and apoptotic pathways. Upon further explorations, the anti-fibrosis underlying mechanism of CA is the increase of α-myosin heavy chain transcription and the inhibition of the fibrotic/hypertrophic markers (CTGF, Collagen Iα, Collagen IIIα, FN/ANP, BNP, and β-MHC) transcription after 4 weeks of treatment. With respect to the decrease of oxidative stress, the study revealed that CA reversed the MI-induced AMPKα/Nrf2/HO-1 pathway inactivation [136]. The nuclear factor-erythroid factor 2-related factor 2 (Nrf2) is a transcription factor that controls the gene expression of the antioxidant response element such as the cytoprotective gene heme oxygenase-1 (HO-1), which degrades antioxidant molecules [137]; the crosstalk of AMPKα and Nrf2/HO-1 pathway was indeed proven by various studies which demonstrated that AMPK activation boosts the Nrf2/HO-1 pathway [138], and, hence, these observations support the above-mentioned anti-oxidative mechanism of CA. The anti-inflammatory effect of CA was supported by its ability to inhibit the mRNA expression of f IL-1β, TNF-α, and IL-6 after myocardial infarction while repressing the expression of NF-κB p65 and p-Ikkβ, an inhibitor of nuclear factor-κB kinase β. Furthermore, CA treatment reversed the myocardial infarction, increased expression of Bax, and decreased expression of Bcl-2, thus revealing its antiapoptotic effect [139].

Doxorubicin is a widely used anticancer drug with severe side effects such as arrhythmias, dilated cardiomyopathy, and heart failure; efforts to inhibit or even decrease its cardiotoxicity led the scientific research efforts towards CA that, as revealed, improved survival rate and cardiac function, restored autophagic flux, promoted mitochondrial biogenesis, reduced the apoptosis, and alleviated the oxidative stress in an in vitro and in vivo doxorubicin-induced cardiotoxicity mice model. Specifically, by activating the AMPKα2/mTORC1 signaling pathway, CA promoted the nuclear translocation of transcription factor EB (TFEB), thus restoring the beneficial autophagic flux which was decreased due to Dox-induced cardiotoxicity. The anti-apoptotic effect of CA was achieved by up-regulating Bcl-2 and down-regulating Bax and caspase-3 expression while the decrease of oxidative stress was a consequence of CA’s ability to inhibit NADPH oxidase Nox 2 and Nox 4 transcription [140].

### 4.4. Other Biological Activities

In liver, AMPK activation regulates gluconeogenesis, glycolysis, and fatty acid oxidation while directly inhibiting and via sterol-regulated element-binding proteins (SREBPs) its downstream targets, acetyl-CoA carboxylase and hydroxy-3-methyl glutaryl coenzyme A reductase, the main enzymes involved in cholesterol and triglyceride synthesis [141,142]. In a tyloxapol (TY)-induced hyperlipidemia mice model, CA was deemed as an anti-hepatic steatosis and antihyperlipidemic agent through a mechanism that involved cholesterol synthesis and inflammation. In detail, the suppression of lipid accumulation induced by CA occurred in a concentration-dependent manner and involved the inhibition of HMGCR, SREBP1c, SREBP2, and SREBP1c target proteins FAS and SCD1 gene expression as well as the activation of AMPK phosphorylation. The anti-inflammatory effect of CA was achieved through ERK, JNK, and p38 MAPK activation and the suppression of IkBα phosphorylation, directly involved in the activation of the NF-kB pathway [143].

A recent study revealed another NF-κB signaling-independent anti-inflammatory mechanism of CA; Kim et al. [144] reported that CA reduced the lipopolysaccharide (LPS)-induced inflammation through the inhibition of IRAK-1 phosphorylation that was previously linked to the suppression of inflammatory cytokines formation. Furthermore, the inhibitory effect was not modified by the addition of IκB-α inhibitors, therefore suggesting the existence of an NF-κB independent mechanism which regulates the anti-inflammatory activity.

## 5. Glycyrrhetinic Acid

**Figure 10:**
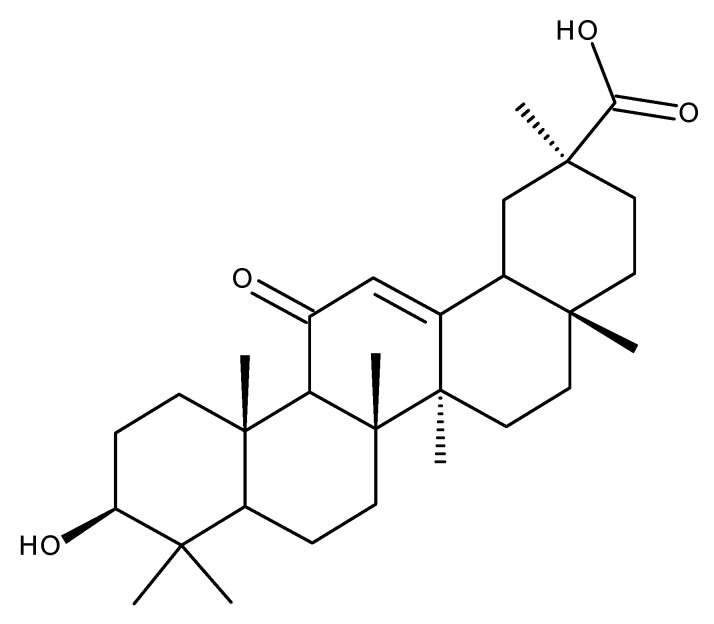
Structure of glycyrrhetinic acid.

**Figure 11:**
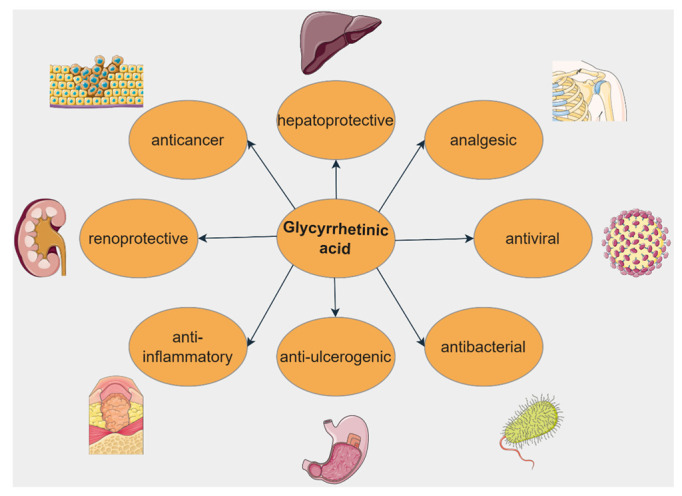
Biological activities of glycyrrhetinic acid. Parts of the figure were drawn by using pictures from Servier Medical Art. Servier Medical Art by Servier is licensed under a Creative Commons Attribution 3.0 Unported License (https://creativecommons.org/licenses/by/3.0/, accessed on 2 August 2022).

Glycyrrhetinic acid (GA. Figure 10) is an oleanane-derived pentacyclic triterpene extracted from the licorice roots (*Glycyrrhiza glabra* and *G. uralensis*), proven to exert numerous beneficial pharmacological activities in various pulmonary, cardiac, arterial, gastric, genitourinary, eye, and other inflammatory diseases (Figure 11) [145,146].

### 5.1. Anticancer Activity

GA was intensely studied in terms of anticancer effects as shown by numerous studies that reported its cytotoxic and pro-apoptotic activity against various types of cancers (Table 3).

### 5.2. Hepatoprotective Activity

**Figure 12:**
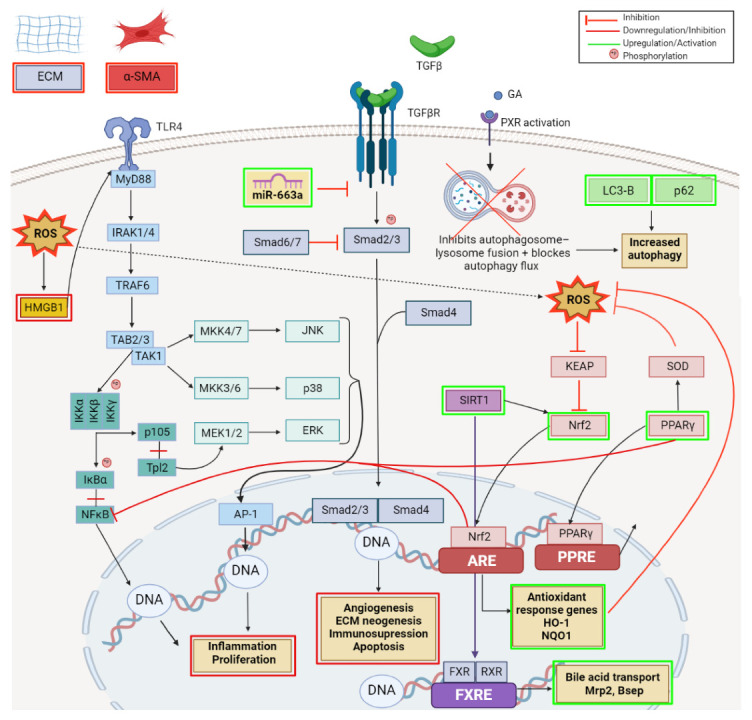
Schematic representation of the reported hepatoprotective mechanisms of glycyrrhetinic acid; key signaling pathways targeted. Created with BioRender.com (accessed on 23 June 2022).

As demonstrated by a vast number of research papers, GA has promising hepatoprotective activity against various types of liver diseases and injuries via multiple mechanisms (Figure 12).

In hepatic stellate (HSC) LX-2 cell line, GA treatment suppressed the activity of HSCs, the extracellular matrix (ECM) production and the alpha-smooth muscle actin (α-SMA) expression [153]. Activation of HSCs and trans-differentiation into activated myofibroblast-like cells that overexpress α-SMA is considered an important step during hepatic fibrosis progression due to their involvement in collagen production; the accumulation of ECM has been recognized as a primary risk factor for the development of portal hypertension, liver cirrhosis, and liver failure [154]. Among the growth factors and pro-inflammatory cytokines, the TGFβ/Smad signaling pathway, modulated by miRNAs, plays a central role in liver fibrosis [155]. The Smad proteins are the intracellular effectors of TGF-β that, once activated by receptors, undergo nuclear translocation where they regulate transcription. As demonstrated by Guo et al. [153], the beneficial effects of GA against liver fibrosis rely on its capacity to upregulate the expression of miR-663a followed by the inhibition of TGF-β/Smad pathway (Figure 10) in HSCs, results also supported by a previous study that indicated miR-663 as a tumor suppressor which acts by regulating the TGF-β1 pathway [156]. An in vitro and in vivo analysis of GA effects in liver injury revealed that GA increases autophagy by upregulating the LC3-B and P62 autophagy-related genes; however, the autophagy increase mainly relies on the activation of the Pregnane X receptor (PXR), a nuclear receptor involved in metabolism, detoxification, inflammation, oxidative stress. and apoptosis with a significant protective effect on liver, thereby inhibiting the autophagosome–lysosome fusion and blocking the autophagy flux (Figure 12) [157].

Several recent studies brought to light the beneficial role of GA and its underlying mechanism of action against drug-induced hepatotoxicity. Specifically, in an acetaminophen-induced hepatotoxicity animal model, GA decreased the hepatic pathological damage and improved transaminase levels by down-regulating the CYP2E1 expression, the main enzyme involved in acetaminophen metabolism; it also inhibited ROS production and deactivated the HMGB1-TLR4 signaling pathway (Figure 12). The high mobility group box 1 (HMGB1), a damage-associated molecular pattern (DAMP) released from necrotic hepatocytes, activates the innate immune system TLR4 signaling pathway. As a consequence, interleukin-1 receptor-associated kinase (IRAK)-1 is phosphorylated and initiates the phosphorylation of JNK, ERK, p38, and IκB, thus causing the activation of downstream signal molecules: nuclear factor kappa B (NFκB) and mitogen-activated protein kinases (MAPKs); hence, the release of excessive inflammatory mediators is stimulated. GA decreased HMGB1 release and inhibited the phosphorylation of IRAK1, IκΒ, JNK, ERK, and p38 (the TLR4 signal pathway) (Figure 12); therefore, the authors concluded that GA can be considered a new approach to treat acetaminophen-induced hepatotoxicity [158]. A similar mechanism of action for GA, involving the inhibition of HMGB1-TLR4 signaling pathway, was also reported by Kuang et al. [159] in a liver ischemia/reperfusion (I/R) injury animal model as well as by Shi et al. [160] in a murine viral hepatitis model.

Another potent drug with limited applications due to its hepatotoxic effects is methotrexate. GA proved to provide hepatic protection against methotrexate-induced hepatic injury by attenuating oxidative stress, inflammation, and apoptosis; the molecular mechanisms involve the up-regulation of Nrf2, a nuclear factor that binds to the antioxidant response element and stimulates the antioxidant protein expression, and PPARγ pathway (Figure 12).

GA ability to prevent drug-induced liver injury and consequent bile acid metabolism disruption has also been reported by Wang et al.; the authors revealed that GA can prevent liver damage induced by alpha-naphthyl isothiocyanate by reversing the alteration of cholesterol metabolism-related mRNAs Cyp7a1, Npc1l1, Mttp, and Acat2 expression, involved in bile acid transport. Moreover, GA stabilized the membrane integrity of mitochondria and lysosomes by inhibiting cathepsin B and thus protecting the liver cell against free fatty acid-induced hepatic lipotoxicity [161]. In a similar ANIT-induced cholestasis rat model, GA activated Sirt1, which regulates bile acid metabolism through deacetylation. This activation consecutively promotes the binding of farnesoid X receptor (FXR) and retinoid X receptor RXR, which upregulates canalicular efflux transporters (Mrp2, Bsep) (Figure 12) and entry into the bile. Simultaneously, Sirt1 activation increases Nrf2 expression, thus leading to the basolateral efflux transporters (Mrp3, Mrp4) upregulation and thereby increasing bile efflux and ameliorating ANIT-induced cholestasis [162].

### 5.3. Anti-Inflammatory Activity

As reported by numerous studies and summarized in a recent review, the anti-inflammatory activity of GA occurs via the induction of the antioxidant defense systems, the decrease of lipid peroxidation, and the alleviation of oxidative and histological damage [163].

In chronic rhinosinusitis, it was found that GA’s anti-inflammatory action is a result of its ability to enhance Sirt6 expression levels which in turn inhibits the nuclear translocation of HMGB1 protein and reverses its extracellular accumulation [164].

Radiation-induced inflammation in RAW264.7 macrophages was alleviated by GA treatment; the results showed that GA decreases ROS production, inflammatory cell infiltration, and TNF-α, IL-1β, and IL-6 levels in cutaneous tissues. Furthermore, the molecular mechanisms appear to involve a decrease in NADPH oxidase activity, the inhibition of NF-κB/p65, and IκB-α phosphorylation, and the decrease of p38MAPK phosphorylation and DNA-binding activity of AP-1 [165]. Similar mechanisms were reported by Zhou et al. [166] in LPS-induced inflammation murine macrophage RAW 264.7 cells; the results showed that the anti-inflammatory activity of GA occurs through the inhibition of NF-κB expression and attenuation of its nuclear translocation. Furthermore, the inhibition of the MAPK/NF-κB signaling pathway by GA was once again demonstrated by an in vitro and in vivo study as the main mechanism of GA anti-inflammatory action in rheumatoid arthritis [167].

In asthma models and bronchial asthma smooth muscle cells GA was proven to inhibit cell proliferation as well as the expression of inflammatory factors by decreasing the mRNA expression of TNF-a, IL-4, IL-6, and YKL-40; upon further examination, the underlying mechanism responsible consisted of the inhibition of ERK1/2 phosphorylation [168].

### 5.4. Antibiotic Activity

The increasing incidence of antiviral and antibiotic drug resistance underlines the need for new effective compounds with limited side effects. In this pursuit, GA has recently emerged as a potent candidate that exerts its beneficial activity through various mechanisms. Pertaining to the molecular mechanisms, in mice infected with *Streptococcus aureus* (SA), GA down-regulated the bacterial-induced HMGB1 expression and inhibited the NF-κB activation, events followed by the subsequent reduction of IL-1β, IL-6, and TNF-α secretion. Thus, GA exhibited anti-inflammatory properties responsible for the increased survival rate of SA-infected mice and the reduced bacterial burden following GA treatment [169]. The results of an in vitro study and an in vivo murine viral hepatitis model demonstrated that the inhibition of HMGB1 pathway is also involved in the GA antiviral activity; GA effectively suppressed the HMGB1 release and its consequent induction of inflammatory cytokines IL-1β, IL-6, IP-10, IL-17A, and IL-22 [160]. Another study on MA104 cells infected with rotavirus (RV) reported that GA has a significant antiviral activity, being able to inhibit the apoptosis of virus-infected cells. The underlying mechanism consisted of the inhibition of the Fas/FasL signaling pathway accompanied by the decrease of caspase 3 and Bax and increase of Bcl-2 expression [170]. Fas is a death receptor located on the cell surface that, upon binding to FasL, is activated and transmits apoptotic signals into the cell.

### 5.5. Antidiabetic Activity

GA was also found to be useful for the prevention and treatment of diabetes; the potential targets of GA antidiabetic activity are currently still under investigation. Studies on insulin resistance found that the culprit behind this key event in type 2 diabetes development is damage in the insulin signaling pathway, more precisely in the phosphorylation of IRS on serine and threonine residues [171]. Normally, the binding of insulin to the α-subunit of the receptor will trigger a conformational change that induces its catalytic activation and consecutive Tyr residues autophosphorylation, leading to the recruitment and phosphorylation of insulin receptor substrate (IRS) and adaptor protein 1 (Shc); the IRS phosphorylation pathway mediates most of the metabolic effects of insulin via the activation of the PI3K/Akt/GSK3β pathway. The other pathway, activated by IRS/Shc, is the Grb2/Sos/Ras/MAPK pathway, which regulates cellular proliferation, differentiation, gene expression, inflammation, apoptosis, and insulin-associated mitogenic effects [172]. A very important aspect is, however, the cross-talk of PI3K⁄Akt and Ras/MAPK pathways activation that seems to be involved in insulin resistance; Zhang et al. reported that GA may improve insulin response via activating both pathways. Specifically, GA inhibited IRS phosphorylation and upregulated AKTser473 and GSK3β expression in the PI3K/Akt pathway while at the same time inhibiting the GTP–Ras binding and consecutive Ras activation; the authors concluded that Ras/MAPK and PI3K/Akt pathway cross-talk is responsible for GA’s antidiabetic effects, namely improved insulin sensitivity, increased cellular glucose consumption level, GLUT4 expression, and reduced inflammation [173].

Another report on the GA putative antidiabetic mechanism in high glucose (HG)-induced THP-1 human monocytic leukemia cell lines revealed that GA inhibited transient receptor potential canonical TRPC3 and TRPC6 protein expressions and the intracellular Ca2+ level [174]. As previously reported, TRPC is a calcium channel protein that regulates cellular proliferation and differentiation; its activation increases intracellular Ca^2+^ level with the control of nicotinamide-adenine dinucleotide phosphate (NADPH) oxidase, inducible nitric oxide synthase (iNOS), and mitochondrial ROS production [175,176]. Furthermore, by inhibiting TRPC, GA decreased the protein expression of iNOS and p47 NADPH oxidase and increased the protein expression of uncoupling protein UCP2, involved in mitochondrial Ca^2+^ and ROS regulation; this cascade of events finally resulted in decreased ROS production and increased sRAGE secretion [174]. sRAGE is the cell-bound receptor of advanced glycation end-products revealed to function as a progression factor that can drive cellular dysfunction that underlies the development of diabetic complications [177]; through its capacity to increase sRAGE secretion, GA exhibits promising antidiabetic potential.

### 5.6. Renoprotective Activity

The renoprotective effects of GA were tested in vitro in a Px-12 thioredoxin inhibitor-induced oxidative injury model of NRK-52E rat renal tubular epithelial cell lines that mimicked chronic kidney disease conditions. It has been reported that thioredoxin-1 inhibitor Px-12 decreased cellular viability, induced morphological changes, increased ROS production, and ultimately induced renal tubular cell oxidative injury by activating the phosphorylation of c-Jun N-terminal kinase (JNK) and, consecutively, by increasing the expression of connexin 43 (Cx43) [178], a gap junction protein recently identified as key player in chronic kidney disease progress [179]. Further, GA treatment was able to alleviate the Px-12-induced oxidative injury by inhibiting JNK phosphorylation and Cx43 expression [178].

### 5.7. Cardioprotective Activity

Upon investigating the GA cardioprotective mechanisms, it was found that in H9c2 myoblasts, a cellular model used as an alternative for cardiomyocytes, GA treatment prevented the apoptosis induced by oxygen glucose deprivation, which mimicked the ischemic heart disease conditions. These beneficial effects proved to be the consequence of GA’s ability to promote the expression of Bcl-2 and to inhibit the expression of Bax and caspase-8 through the PI3K/Akt pathway [180]. Similar inhibitory effects of the PI3K/Akt pathway were described by another study to be the cardioprotective mechanism of GA; Chu et al. [181] reported that GA alleviates oxidative stress, inflammation, and apoptosis in a mice model of myocardial infarction via PI3K/Akt pathway inhibition while also decreasing Ca^2+^ influx through L-type calcium channels, thus reducing cell contractility. The GA effect on Ca^2+^ was also described in an ischemia-reperfusion rat model that demonstrated the significant decrease of intracellular Ca^2+^ levels in cardiomyocytes through the inhibition of the late sodium current, thus improving diastolic function in patients with heart failure [182].

### 5.8. Other Biological Activities

Several literature data have revealed GA’s beneficial properties and mechanisms of action in other various pathological conditions. In antibiotic-induced intestinal injury, characterized by decreased cell proliferation, cell cycle arrest, and delayed wound healing, GA reversed the antibiotic-induced inhibition of proliferation and G0/G1 phase arrest by upregulating cyclin D1 expression. Moreover, the decreased expression levels of human antigen R (HuR) (i.e., RNA binding protein involved in intestinal proliferation and regeneration after injury) observed after antibiotic treatment were restored following GA treatment, suggesting that GA can maintain intestinal homeostasis post-transcriptionally through HuR restauration [183].

A recent paper demonstrated that GA may extend its beneficial effects even at the level of the nervous system. Against realgar (a mineral drug-containing arsenic)-induced neurotoxicity, the neuroprotective effects of GA occur via the activation of Nrf2 pathway that in turn increases the transcription of the downstream corresponding target genes HO-1, xCT, EAAT3, MRP-1, and γ-Glutamylcysteine synthetase GCLC and GCLM. Moreover, GA also increased the brain supply levels of Glu and Cys and the expression of γ-Glutamyl transpeptidase (γ-GT); thus, GA restored the hippocampal brain glutathione (GSH) levels that were depleted in the context of realgar administration and provided neuroprotection [184].

Moving into the investigation of GA’s protective mechanism against acute lung injury, Wang et al. reported its involvement in the modulation of the PI3K/Akt pathway. More precisely, GA decreased ROS production and downregulated the macrophage Nod-like receptor 3 (Nlrp3) inflammasome activation, a major component of innate immunity, via decreasing the phosphorylation level of PI3K and AKT in macrophages [185].

## 6. Madecassic Acid

**Figure 13:**
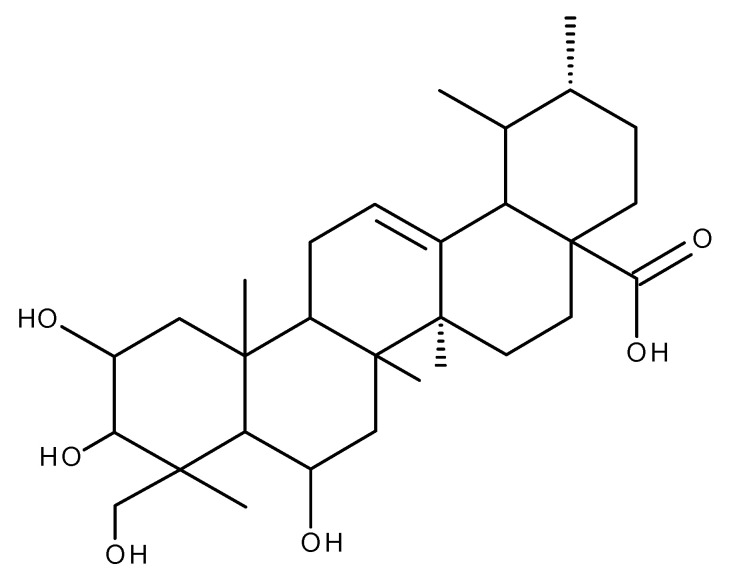
Structure of madecassic acid.

Madecassic acid (MdA, Figure 13) is one of the four main compounds extracted from *Centella asiatica* in addition to asiatic acid and triterpene glycosides asiaticoside and madecassoside. Its biological activity spectrum includes antioxidative, antidiabetic, and hypolipidemic effects [186]. Additionally, Xu et al. [187] reported that MdA was able to ameliorate colitis induced by dextran sulfate sodium (DSS) in mice. The excessive immune system activation in colitis occurs mainly due to Th17/Treg imbalance, more precisely to the increase of functionally active Th17 cells that cause autoimmunity and inflammation in parallel with a lack of immune-suppressive Treg cells [188]. Furthermore, MdA restores the balance of Th17/Treg via AMPK activation that in turn downregulates acetyl CoA carboxylase (ACC1) expression, an enzyme that catalyzes the carboxylation of acetyl-CoA to malonyl-CoA in fatty acid synthesis and enhances and inhibits the polarization of Th17 and Treg cells [189]. Furthermore, MdA increased the expression of CD36 and LPL in the colon of DSS-induced mice, two PPARγ-responsive genes; it also induced nuclear translocation of PPARγ and the binding of PPARγ to a reporter. Additional testing revealed that AMPK is a downstream effector of PPARγ and the study concluded that MdA is a PPARγ agonist that regulates AMPK/ACC1-mediated Th17/Treg balance [187].

## 7. Maslinic Acid

**Figure 14:**
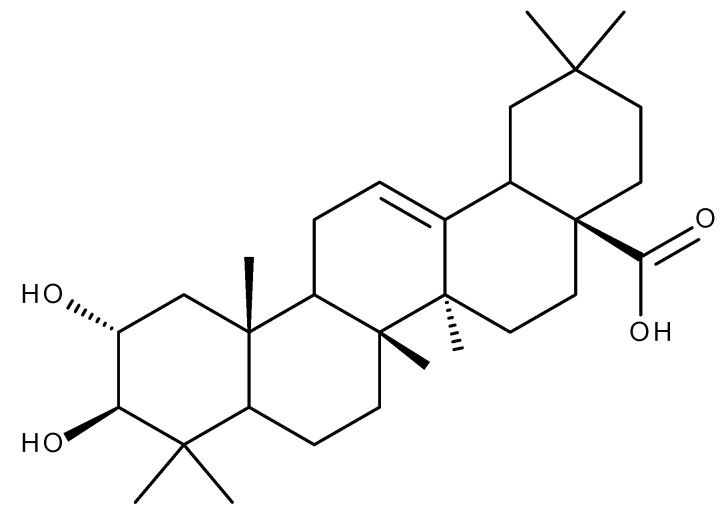
Structure of maslinic acid.

**Figure 15:**
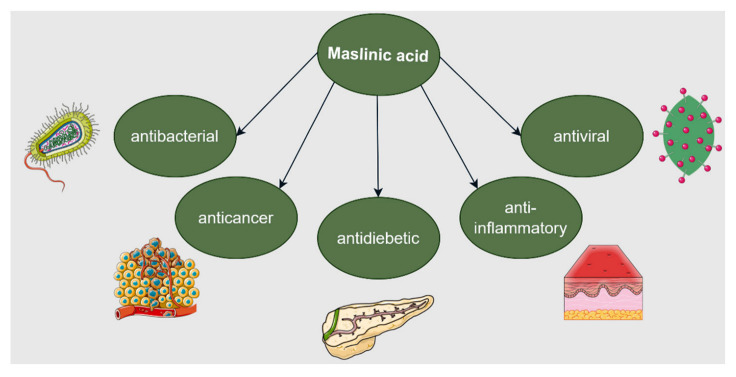
Biological activities of maslinic acid. Parts of the figure were drawn by using pictures from Servier Medical Art. Servier Medical Art by Servier is licensed under a Creative Commons Attribution 3.0 Unported License (https://creativecommons.org/licenses/by/3.0/, accessed on 2 August 2022).

Maslinic acid (MA, Figure 14), extracted mainly from olives (*Olea europaea*), has attracted the interest of scientific research due to its plethora of therapeutic properties such as anti-inflammatory, antimicrobial, antiviral, antidiabetogenic and anticancer effects (Figure 15) [190].

### 7.1. Anticancer Activity

As reported by a large number of studies, MA exhibits antitumor effects in various types of cancer such as breast, colorectal, melanoma, and lung [191]. Many of these studies have demonstrated MA’s ability to inhibit cell proliferation and to induce apoptosis; however, its beneficial activities extend beyond those presented and rely on various mechanisms as presented in Table 4.

### 7.2. Anti-Inflammatory Activity

The recent study of Shimazu et al. followed the investigation of MA’s underlying anti-inflammatory mechanism in collagen antibody-induced arthritis mice models; MA suppressed inflammation by decreasing the secretion of IL-6 and TNF-α, a consequence of MA’s ability to inhibit the NF-κB signaling pathway. The same group also revealed that MA can inactivate the Toll-like receptor 4 (TLR4) receptor, an upstream regulator of NF-κB. Furthermore, MA altered the expression levels of genes that are controlled by the glucocorticoid receptor (“the glucocorticoid receptor signaling”), leading to a reduced production of LTB4, a chemotactic factor and neutrophil activator, highlighting once again the anti-inflammatory action of MA [201].

### 7.3. Cardioprotective Activity

Atherosclerosis, the main underlying cause of ischemic heart disease, is a chronic progressive event characterized by the accumulation of cholesterol in the main large–medium-sized arteries followed by plaque formation. The immense number of deaths attributed to ischemic heart disease put atherosclerosis at the center of many studies that tried to elucidate its pathophysiology in order to employ the most effective treatment options. Taking this into account, it was proven that inflammation is the key element in atherosclerosis pathogenesis [202,203], accompanied by oxidative stress. Turning to the study of natural compounds as potential adjuvant or treatment options, the documented anti-inflammatory and anti-oxidative effect of MA led to its assessment for potential anti-atherosclerotic properties. In this regard, Phang et al. demonstrated that MA targets important steps of plaque formation such as cholesterol influx, LDL peroxidation, monocyte adhesion, and, finally, foam cell formation. In detail, MA significantly inhibited vascular cell adhesion molecule-1 (VCAM-1) and monocyte chemoattractant protein-1 (MCP-1) protein expressions via the inhibition of TNF-α, shown to have pro-atherosclerotic effects on endothelial cells. Within the atherosclerotic oxidative processes, MA decreased the CuSO_4_-induced lipid peroxidation while also inhibiting the protein expression of SR-A and CD36, two regulating scavenger receptors (that uptake the oxidized lipoproteins and lead to foam cell formation) [204].

Upon myocardial infarction treatment, the reperfusion process itself may also lead to irreversible damage to the myocardium, generally termed ischemia/reperfusion injury (I/R); the principal players involved in the development of I/R injury are inflammation and apoptosis, both associated with the TRL4/NF-κB signaling pathway [205,206]. In vitro and in vivo testing revealed that MA can indeed alleviate the I/R injury by inhibiting inflammation and apoptosis via HMGB1/TLR4/NF-kB pathway inhibition [207]. Similar involvement of the NF-kB pathway in MA cardioprotective mechanisms was reported by Ampofo et al.; MA inhibited the hypoxia-activated NFκB pathway by decreasing the IκBα phosphorylation that, in turn, decreased its nuclear translocation and the phosphorylation and DNA-binding of p65. This inhibitory effect led to the consecutive decrease of surface adhesion proteins E-selectin, VCAM-1, and ICAM-1 levels in endothelial cells, thus reducing inflammation after I/R. In addition, within the same experimental settings, MA significantly increased the expression of endothelial nitric oxide synthase (eNOS) which, in turn, decreased the ROS and cytokines production [208].

### 7.4. Antidiabetic Activity

Mwakalukwa et al. [209] conducted a recent study in order to determine the antidiabetic potential of seven compounds extracted from the olive mill wastes; by analyzing the inhibitory kinetics of the active compounds against α-glucosidase and α-amylase enzymes, the authors concluded that MA exerts high inhibitory activity against both enzymes, thus concluding that MA is a potential tool to control postprandial hyperglycemia and to prevent diabetes.

### 7.5. Neuroprotective Activity

Maslinic acid was found useful as a treatment against cognitive dysfunction induced by cholinergic blockade in vivo in animal models; experimental results showed that the phytocompound was able to activate TrkB receptor by binding it to mBDNF (brain-derived neurotrophic factor), leading to the activation of PI3K/Akt and ERk-CREB pathways, hence improving cognitive deficits [210]. BDNF is one of the major protein regulators of biological functions in the nervous system, being synthetized as a precursor proBDNF that suffers proteolytic cleavage in order to become a mature molecule; the proBDNF/MBDNF ratio is determined by neuronal activity [211]. The property to ameliorate loss of skeletal muscle and strength of maslinic acid was examined by Yamauchi et al.; in terms of molecular mechanism, MA regulated muscle synthesis through upregulating IGF-1 expression and downregulating TGF-β and NF-kB pro-inflammatory cytokines as a result of TNFα signaling pathway inhibition, thus leading to a mediation of inflammatory effects. Furthermore, the phytocompound alleviated muscle atrophy by suppressing Atrogin-1 and Murf-1 atrophic genes expression [212].

### 7.6. Hepatoprotective Activity

The hepatoprotective effect of MA is achieved through the inhibition of NF-κB and stimulation of Nf-2 pathways which lead to decreased levels of TNF-α and IL-6 pro-inflammatory cytokines and increased concentrations of antioxidant HO-1 enzyme [213]. Moreover, MA exerted beneficial effects in NALFD through the activation of the Sirt1/AMPK signaling pathway, which augmented lipolysis and diminished lipogenesis both in vivo and in vitro [214].

### 7.7. Renoprotective Activity

In an in vitro study conducted to assess the effect and underlying mechanism of MA’s activity in renal fibrosis, MA downregulated MyD88 and TGF-β/Smad pathways, leading to the decrease of extracellular matrix proteins expression and pro-inflammatory cytokines, thus inhibiting renal fibroblast proliferation [215].

### 7.8. Other Biological Activities

MA could be employed in the treatment of *Acanthamoeba keratitis*, an infection produced by the genus Acanthamoeba, due to its amoebicidal effect on both trophozoite and cyst stages of the protozoa; MA acts through apoptosis induction as confirmed by the morphological changes of the protozoa cells [216]. However, the study did not provide information regarding the underlying molecular mechanism, which remains to be further investigated.

## 8. Pomolic Acid

**Figure 16:**
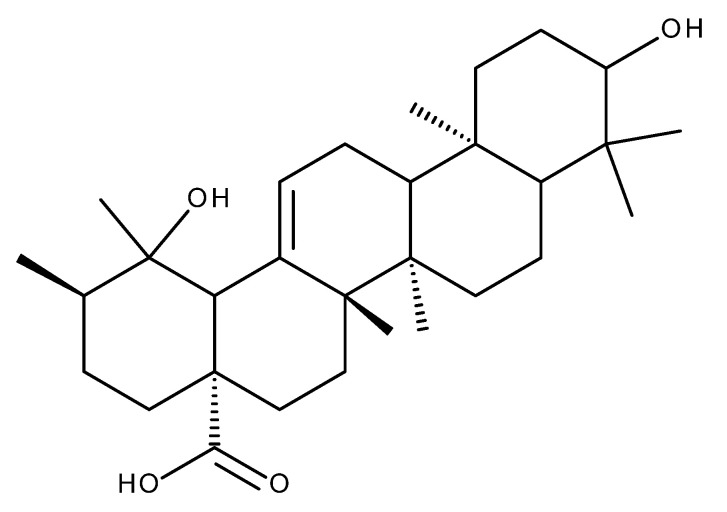
Structure of pomolic acid.

Pomolic acid (PA, Figure 16) is an ursane-type triterpenoid found in low quantities in various sources such as apples peels, rosemary (*Rosmarinus officinalis*), loquats (*Eriobotrya japonica*), and coco plum (*Chrysobalanus icaco*) [217]. In terms of biological activity, PA mainly exhibits an anticancer effect; however, several early studies reported hypotensive, platelet anti-aggregating, and antiviral effects [218,219].

Earlier investigations reported cytotoxic effects and cell migration inhibition after PA treatment against breast cancer, human gastric, ovarian and uterine adenocarcinoma, murine melanoma, and leukemia cell lines [220,221,222,223]. Recent attempts to explore the mechanisms for its anticancer effectiveness revealed PA’s ability to trigger apoptosis via several pathways in prostate cancer, glioma, and leukemia cell lines. Guimarães et al. tested PA’s effect in A172, U87, and GBM-1 human glioblastoma cells and revealed decreased cellular viability and cell migration as well as increased cell apoptosis after PA treatment, as demonstrated by MTT and DNA fragmentation assays. Specifically, PA treatment increased caspase-3 and -9 activity and ROS production while decreasing the mitochondrial transmembrane potential in a time-dependent manner; the results indicate that the PA pro-apoptotic mechanism of action involves the activation of the intrinsic pathway in the presence of increased ROS production. The inhibition of glioblastoma cell migration in the same study was revealed to be a consequence of PA’s ability to down-modulate the activity of MRP1/ABCC1, two important transporter proteins involved in tumor drug resistance and progression [224]. Martins et al. investigated the molecular mechanisms behind the antitumor activity of PA in parental PC3 and docetaxel-resistant prostate cancer PC3R cell lines and revealed a decrease in cell viability accompanied by cell apoptosis. The results showed that PA has similar effects on PC3 and PC3R, thus indicating that the compound can bypass the resistance mechanisms induced by docetaxel (DTX); PA did not interfere with the activity of P-gp/ABCB1 in the PC3R cell line, where the inhibition of this pathway seems to revert its resistance to DTX. However, PA down-modulated MRP1/ABCC1 activity in both PC3 and PC3R cell lines, thus suggesting that this mechanism is responsible for PA’s cytotoxicity observed in both cell lines [225]. In acute myeloid leukemia cell lines HL60, U937, and Kasumi-1, PA decreased cell viability and cell growth and induced cell death via the activation of the caspase pathway and inhibition of hTopo I and IIα human DNA topoisomerases in all cell lines [10].

## 9. Conclusions and Future Perspectives

Cancer, diabetes, and inflammatory diseases have become, among many others, unmanageable and difficult to treat. Not only do classical therapies cause a wide range of unbearable side effects, but they are the root for several associated pathologies that altogether makes the patient’s life very difficult. The urge to discover wonder treatments has become crucial; hence, a plethora of researchers have dedicated their lives to examine different types of compounds in order to find suitable alternatives against these uncurable pathologies.

Avoiding the great extent of side effects represents the main selection criteria; hence triterpenoid compounds might offer the answer for future treatments that could improve patients’ compliance. Triterpenoid compounds possess a wide spectrum of pharmacological activities, including anti-cancer, antiviral, anti-inflammatory, antidiabetic, cardioprotective, neuroprotective, and hepatoprotective effects, etc. Their potential has been extensively investigated in the last decades; furthermore, the molecular mechanisms responsible for their pharmacological activities have been greatly clarified in order to establish proper formulations for future clinical trials.

Our study aimed to outline the molecular mechanisms of several widely explored triterpenoid acids, namely, BA, BoA, CA, GA, MA, MdA, and PA. The literature study of these representants revealed a wide spectrum of molecular pathways involved in triggering different types of pharmacological responses, being tested in vitro, in vivo and in ovo. These current findings might represent the foundation for consolidating future research and implementing triterpenoid compounds as valid alternative therapies to classical ones.

## Figures and Tables

**Table 1:** In vitro and in vivo biological activity of betulinic acid against different types of cancers.

Type of Cancer	Experimental Conditions	Mechanism	Outcome	Reference
Glioblastoma	In vitro—U87MG and A172 cell lines	- Suppressed the NF-κB pathway - Downregulated the levels of pro-survival pathway factors: survivin and XIAP - Up-regulated the expression of caspase-3 and caspase-9	- Induced glioblastoma apoptosis	[24]
Oral squamous carcinoma	In vitro—OSCC-derived cell line KB In vivo—KB injected Balb/c nude mice	- Increased ROS production which led to mitochondrial apoptosis - Stimulated the expression of caspase-3, caspase-9 and Bax/Bcl-2 ratio	- Induced mitochondrial apoptosis, cell cycle arrest and inhibition of cell proliferation, which further led to tumor reduction	[25]
	In vitro—CAL-27 and Tca-83 oral squamous carcinoma cell lines	- Modulated the Sp1/PTEN pathway - Stimulated the SP1 production, leading to PTEN overexpression - PTEN inhibited PI3K/Akt and TNF-α/ NF-κB pathways	- Promoted cell apoptosis and inhibition of cell proliferation	[26]
Gastric cancer	SGC-7901 drug-resistant cancer cell lines	- Downregulated ERK/MEK signaling pathway - Inhibited the phosphorylation of ERK and MEK proteins	- Induced autophagy and stimulated the autophagosome formation	[27]
Hepatocarcinoma	In vitro—HepG2 and SMMC-7721 hepatocarcinoma cell lines	- Increased the Bax/Bcl-2 ratio and caspase-3 activity - Decreased the p62 levels and stimulated the LC3B-II and beclin-1 levels - Down-regulated the PI3/AKT/mTOR signaling pathway	- Induced apoptosis and autophagy and inhibited cancer cell proliferation	[28]
	In vitro—PLC/PRF/5 and MHCC97L hepatocarcinoma cells	- Inhibited the expression of RNA MALAT1 - Increased the expression of miR-22-3p, leading to degradation of IAPs	- Induced cell apoptosis	[29]
Pancreatic cancer	In vitro—SW1990 and PANC-1 pancreatic carcinoma cell lines In vivo—mouse xenograft model	- Inhibited the mTOR pathway - Upregulated the p-AMPK expression, leading to inhibition of protein synthesis	- Inhibited malignant cells proliferation, stimulated autophagy and reduced tumor growth	[30]
Acute myeloid leukemia	In vitro—Kasumi-1, HL-60 and THP-1 leukemia cell lines	- Inhibited SOD-2 levels, leading to ROS generation - Activated AHR receptor and HIF-1α level	- Inhibited cell proliferation and enhancing apoptosis	[34]
	In vitro—u937 leukemia cell line	- Increased the Bax/Bcl-2 ratio, caspase-3, caspase-9 levels - Induced PARP degradation - Upregulated p21WAF1/CIP1 and downregulated cyclin A and B1 levels	- Induced apoptosis and G2/M phase cycle arrest	[35]
Non-small lung cancer	In vitro—A549, H358 and NCI-H1703 cell lines	- Increased expression of p21 and decrease expression of cyclin D1 and B1 - Increased Bax/Bcl-2 ratio, caspase-3 and caspase-7 levels - Activated ERK	- Induced G1 cell cycle arrest and cancer cell apoptosis	[37]
Breast cancer	In vitro—MDA-MB-231 cells	- Decrease the expression of Bcl-2 and increased Bac/Bcl-2 ratio	- Induced cellular apoptosis and inhibited cancer cell proliferation	[38]
	In vitro—MDA-MB-231 cells	- Enhanced PERK binding to GRP78 receptors, leading to PERK accumulation - Initiated the phosphorylation of elF2α, leading to β-catechin and c-Myc-mediated glycolysis inhibition	- Inhibited anaerobic glycolysis and cancer cells metastasis	[39]
Ovarian cancer	In vitro—A2780 ovarian cancer cells	- Down-regulated Bcl-2 levels, up-regulated Bax levels and induced caspase-3, -8, and -9 - Stimulated ROS generation and DNA damage	- Inhibited cancer cell proliferation and enhanced apoptosis	[40]
Cervical cancer	In vitro—HeLa cells	- Inhibited Thr308 and Ser473 phosphorylation, leading to the production of ROS - Suppressed PI3K-Akt pathway - Enhanced expression of p21, p27, and caspase-9 pro-apoptotic factors	- Enhanced cancer cells apoptosis	[41]
	In vitro—HeLA cells	- Degraded HIF-1α by activating β1, β2, and β5 proteasomes, leading to the inhibition of VEGF, GLUT1, HK and PDK1 gene expression	- Inhibited tumorigenesis	[42]
Bladder cancer	In vitro—T-24, UMUC-3 and 5637 human bladder cancer cell lines	- Inhibited cyclin A, cyclin B1m CDK-2, CDC-2, and Cdc25c levels - Up-regulated BAX levels, leading to caspase-8, -9, and -3 activation and increased PARP concentrations - Inhibited Snail, Slug, and MMP-9 levels	- Induced G2/M cell cycle arrest - Induced cancer cells apoptosis	[43]
	In vitro—EJ and T24 human bladder cancer cell lines	- Inhibited the phosphorylation of mTOR and ULK1 - Stimulated AMK phosphorylation	- Enhanced autophagy by stimulating autophagosomes formation	[44]
Colorectal cancer	In vitro—HCT116 cell line	- Down-regulated WNT, HIF-1α and EGRF - Up-regulated p53, Myc/Max, TGF-β, and caspase-9 levels	- Inhibited the angiogenesis and tumor cells proliferation	[45]
	In vitro—HCT116 cell line In vivo—mouse model	- Increased Bax/Bcl-2 ratio and caspase-9 levels - Decreased MMP expression and increased TIMP-2 expression - Enhanced ROS formation	- Induced mitochondrial apoptosis	[46]
	In vitro—HCT116 cell line	- Increased p53 expression initially - Afterwards, it induced the ubiquitin-mediated degradation pathway, leading to p53 catabolization	- Induced autophagy	[47]

**Table 2:** In vitro and in vivo biological activity of corosolic acid against different types of cancers.

Type of Cancer	Experimental Conditions	Mechanism	Outcome	Reference
Gastric cancer	In vitro	- Activated the AMPK pathway - Stimulated AMPK phosphorylation - Restored sensitivity of 5-fluorouracil gastric cancer	- Decreased 5-fluorouracil resistant gastric cancer	[116]
		- Up-regulated Bax and IκB-α expressions, leading to NF-κB transcription inhibition - Down-regulated the expression of p65, IκB-α, Fas, smac, and Bcl-2	- Inhibited gastric cells proliferation and enhanced	[117]
Colorectal cancer	In vitro—HCT116 and SW480 colorectal cancer cell lines In vivo—*colon carcinoma xenograft model*	- Inhibited the heterodimerization and phosphorylation of HER2 and HER3 - Inhibited the PI3K/Akt and Ras/Raf/MAPK signaling pathways	- Produced mitochondrial dynamics changes, leading to apoptosis	[118]
Liver cancer	In vitro—s Bel-7404, HL-7702, Bel-7402, SMMC-7721, SKHep1, and HEK-293T liver cancer cell lines In vivo—xenotransplantation model	- Inhibited the activity of CDK19, leading to inhibiting the (YAP)-O-GlcNAcylation pathway - Reduced the expression of YAP and OGH	- Decreased cell proliferation and reduced tumor growth	[120]
	In vitro—Hep3B and HepG2 hepatocellular carcinoma cell lines In vivo—xenotransplantation model	- Reduced the transcription of Runx2 and TEAD by promoting the of binding YAP to CREB - Promoted the translocation of YAP from the nucleus in HCC	- Inhibited cell proliferation and tumorigenesis	[121]
Rectal cancer	In vitro—ACHN and A498 rectal cancer cell lines	- Increased ROS production leading to lipid peroxidation - Induced caspase-independent non-apoptotic cell death	- Induced lipid peroxidation-dependent non-apoptotic cell death	[122]
Prostate cancer	In vitro —PC-3 and DU145 prostate cancer cell lines In vitro—xenograft tumor model	- Increased the Bip expression, leading to ER stress activation - Activated the IRE-1/ASK1/JNK pathway and the PERK/eIF2α/ATF4/CHOP pathway, leading to p-AKT inhibition and Bax protein overexpression	- Inhibited cell growth, induced cancer cell apoptosis	[123]
Glioblastoma	In vitro—GBM8401, M059K and U-87MG malignant glioma cell lines	- Down-regulated the AXL pathway, leading to the inhibition of JAK2/MEK/ERK axis - Up-regulated the CHIP protein expression, an upstream regulator of AXL pathway	- Inhibited cell proliferation and promoted apoptosis	[124]
Retinoblastoma	In vitro—Y-79 and ARPE-19 human retinoblastoma cell lines	- Inhibited the (MELK)-FoxM1 signaling pathway by inhibiting MELK and FoxM1 expressions	- Induced cellular apoptosis	[125]

**Table 3:** In vitro and in vivo biological activity of glycyrrhetinic acid against different types of cancers.

Type of Cancer	Experimental Conditions	Mechanism	Outcome	Reference
Ovarian cancer	In vitro—Human umbilical vein endothelial cells (HUVECs) and A2780 human ovarian cancer cells Ex vivo and in vivo models: rat aortic ring assay, zebrafish, and mouse Matrigel plug assay	- inhibited the phosphorylation of VEGFR2, mTOR, Akt, ERK1/2, MEK1/2, p38, and JNK1/2 in HUVECs - increase of p38 and JNK1/2 phosphorylation, cleavage of caspase 3, caspase 9, and PARP - reduction of mTOR, Akt and ERK1/2 phosphorylation and survivin and cyclin D1 expressions in A2780 ovarian cancer cell - suppression of VEGF-induced microvessel sprouting in rat aortic ring model	- inhibited proliferation, migration, invasion, and tube formation in HUVECs - induced apoptosis, loss of mitochondrial membrane potential and cell cycle arrest in G1 phase in A2780 ovarian cancer cell - decreased the size of tumors in xenograft mice—inhibition of new blood vessel formation in zebrafish model - the in vitro antiangiogenic and proapoptotic were confirmed in ex vivo and in vivo models.	[145]
Hepato-cellular carcinoma (HCC)	In vitro—hepatocellular carcinoma HepG2, SMMC-7721, HLF, HLE, LM3, and Hep3B cell lines In vivo—xenograft tumorigenicity assay	- inhibition of the cyclin D1 and cyclin-dependent kinase (CDK)4 expression—cell cycle-related proteins - promoted the Microtubule-associated protein 1A/1B-light chain 3 (LC3B) accumulation, a hallmark of autophagy - increase the expression of unfolded protein response (UPR): ATF4, CCAAT-enhancer-binding protein homologous protein (CHOP), IRE-1α, and X-box binding protein (XBP)-1s in SMMC-7721 and HepG2 cell lines - induction of endoplasmic reticulum stress in HCC cells that activated unfolded protein response (UPR): ATF4/CHOP and IRE-1α/XBP1s pathways	- reduction of HCC cell proliferation in a dose-dependent manner in vitro; - induce G0/G1 arrest in three HCC cancer cell lines in a dose-dependent manner; promoted tumor cell death by apoptosis - induced autophagy in vitro and in vivo in HCC cells - ATF4/CHOP signaling pathway induced cytoprotective autophagy and apoptosis, while IRE-1α contributed to survival of HCC cells	[147]
Gastric cancer	In vitro—human gastric cancer cell line SGC-7901	- decrease of MMP-2 and 9 activity, two enzymes involved in the epithelial mesenchymal transition (EMT) process - upregulation of E-cadherin expression, a tumor suppressor gene that can inhibit EMT and reduce cancer metastasis - downregulation of vimentin expression that maintains the cellular ultrastructure integrity - inhibition of PKC-α and the activation of ERK in a dose-dependent manner	- cellular viability suppression - reduction of cell invasion and migration - decrease of intracellular ROS formation - inhibition of gastric cancer metastasis	[148]
Prostate cancer	In vitro—LNCaP, PC3, DU145, human prostate cancer cells	- induction of miR-488 expression, a tumor suppressive microRNA, that resulted resulting in the down-regulation of androgen receptor (AR) and CDK2 expression - transcriptional down-regulation of AR by controlling E2F3α and SRF function on the AR promoter - inhibition of cellular responses mediated by androgens - suppression of androgen target genes (TMPRSS2, PSA, and NKX3.1) expression	- induction of cell death in a dose-dependent manner - inhibition of androgenic and survival responses of LNCaP cells	[149]
Colorectal cancer	In vitro—LoVo, SW480 and SW620 colorectal cancer cells In vivo—xenograft tumor models	- reduction of survivin expression - induction of cleaved PARP expression - MMP expression downregulation - inhibition of PI3K/AKT, signal transducer and activator of transcription 3 (STAT3), p38, JNK, and NF-κB phosphorylation	- inhibition of colorectal cancer cell survival and proliferation in a dose- and time-dependent manner - apoptosis induction - reduction of wound healing capability, cell migration and invasion	[150]
Lung cancer	In vitro—A549 lung cancer cells, IMR-90 human embryonic lung fibroblasts	- upregulation of E-cadherin expression in A549 cells - downregulation N-cadherin, vimentin, and SNAI 1 expression in A549 cells - increase of intracellular ROS levels in A549 cells and decrease of IMR-90 ROS levels - increase of p-ERK, p-STAT3, NF-κB, and Bcl-2 expression levels - activation of ROS/MAPK/STAT3/NF-κB signaling pathway	- induction of ROS/mitochondrial-dependent apoptosis - G2/M cell cycle arrest - inhibition of cell migration	[151]
Sarcoma	In vitro—HOS human osteosarcoma cells and HT1080 human fibrosarcoma cells In vivo—xenograft osteosarcoma models	- downregulation of Cyclin E, CDK4, and Cyclin D expression - increase of JNK and c-Jun phosphorylation - increase of caspase-3, -8, and -9 and PARP activity - reduction of Bcl-2, Bcl-xl, and survivin expression	- induction of G0/G1-phase arrest - autophagy induction via JNK/c-jun pathway activation - inhibition of cell proliferation - induction of apoptosis through both intrinsic and extrinsic pathways and cell death	[152]

**Table 4:** In vitro and in vivo biological activity of maslinic acid against different types of cancers.

Type of Cancer	Experimental Conditions	Mechanism	Outcome	Reference
Breast carcinoma	In vitro—docetaxel-resistant MDA-MB-231 breast carcinoma cells	- suppressed the expressions and interaction of MELK and FoxM1 - decreased the transcriptional activity of FoxM1 and consequently reduced the expression of ABCB1 (ATP binding cassette subfamily B member 1), a gene that encodes a membrane-associated protein of the ATP-binding cassette (ABC) family	- decreased docetaxel resistance - increased docetaxel cellular accumulation - decreased cellular viability	[192]
Cervical cancer	In vitro—HeLa human cervical cancer cells	- increased the levels of p-ATMSer1981, p-ATRSer428, p53, p-p53Ser151, and p-H2A.XSer139, protein kinases involved in DNA repair - increased the levels of BRCA1 and PARP - decreased the levels of DNA-dependent protein kinase, a mediator of the cellular response to DNA damage - decreased the levels of MGMT that normally is involved in DNA adducts repairs at the O6 position of guanine	- decreased cellular viability - induced DNA condensation, fragmentation and damage	[193]
Colorectal cancer	In vitro—HT-29, HCT 116, SW480, SW48, and LS 174T colorectal cancer cells	- down-regulated cyclin D1 - increased IκK-β and consequently decreased the transcriptional activation of NF-κB	- inhibited cellular growth - induced cell cycle arrest - produced apoptosis via NF-κB pathway inhibition	[194]
Colorectal cancer	In vitro—HCT116 and SW480 colorectal cancer cells In vivo—mice and xenograft tumor model	- increased the phosphorylation of AMPK - activated the AMPK pathway partly by regulating AMP and ATP levels - decreased mTOR phosphorylation and consequently downregulated the phosphorylation of 4EBP1 and p70S6K; a down-stream translation repressor protein (4EBP1) and a mitogen-activated Ser/Thr protein kinase (p70S6K) that is required for cell growth	- decreased cellular proliferation, migration, tumor growth and tumorigenesis - promoted apoptosis - induced cell cycle arrest at the G2 Phase	[195]
Gastric cancer	In vitro—MNK28 human gastric cancer cell	- decreased Bcl-2/Bax expression level - inhibited IL-6-mediated STAT3 activation - inhibited the JAK/STAT3 pathway	- inhibited cell viability and proliferation - induced apoptosis	[196]
Neuroblastoma	In vitro—SHSY-5Y Human neuroblastoma cell line	- increased Bax expression - induced caspase-3 and caspase-9 activation - inhibited MAPK/ERK signaling pathway	- decreased cell viability - inhibited cell migration and invasion - altered the cellular morphology - decreased ROS production - induced apoptosis	[197]
Pancreatic cancer	In vitro—Panc-28 pancreatic cancer cells	- down-regulated the expression of HSPA8 (heat shock protein family A (Hsp70) member 8); its role is to bind to nascent polypeptides in order to facilitate correct protein folding - HSPA8 down-regulation lead to decreased mTOR phosphorylation which in turn increased the expression of p-ULK1l Atg3, Atg5, Atg16L, Atg7, and Atg12 (autophagy related proteins) and the ratio of LC3-II/LC3-I	- inhibited cell viability - induced apoptosis - induced autophagy via HSPA87 down-regulation	[198]
Pancreatic cancer	In vitro—PANC-1 and Patu-8988 pancreatic cancer cells	- down-regulated uveal autoantigen with coiled-coil domains and ankyrin repeats (UACA) gene and protein expression - down-regulated adenylate kinase 4 (AK4) gene and protein expression	- inhibited cell viability, migration, and invasion - induced apoptosis	[199]
Pheochromocytoma	In vitro—PC12 rat adrenal pheochromocytoma cells	- promoted LC3-I/II conversion, thus initiating the formation of the autophagosome - blocked the interaction of Bcl2-Beclin1	- induced autophagy	[200]
